# Switchable Nitroproteome States of *Phytophthora infestans* Biology and Pathobiology

**DOI:** 10.3389/fmicb.2019.01516

**Published:** 2019-07-16

**Authors:** Karolina Izbiańska, Jolanta Floryszak-Wieczorek, Joanna Gajewska, Jarosław Gzyl, Tomasz Jelonek, Magdalena Arasimowicz-Jelonek

**Affiliations:** ^1^Department of Plant Ecophysiology, Faculty of Biology, Adam Mickiewicz University in Poznań, Poznań, Poland; ^2^Department of Plant Physiology, Faculty of Horticulture and Landscape Architecture, Poznań University of Life Sciences, Poznań, Poland; ^3^Department of Forest Utilization, Faculty of Forestry, Poznań University of Life Sciences, Poznań, Poland

**Keywords:** protein tyrosine nitration, peroxynitrite, reactive nitrogen species, pathogen offensive strategy, *Phytophthora infestans*, potato, late blight

## Abstract

The study demonstrates protein tyrosine nitration as a functional post-translational modification (PTM) in biology and pathobiology of the oomycete *Phytophthora infestans* (Mont.) de Bary, the most harmful pathogen of potato (*Solanum tuberosum* L.). Using two *P. infestans* isolates differing in their virulence toward potato cv. Sarpo Mira we found that the pathogen generates reactive nitrogen species (RNS) in hyphae and mature sporangia growing under *in vitro* and *in planta* conditions. However, acceleration of peroxynitrite formation and elevation of the nitrated protein pool within pathogen structures were observed mainly during the *avr P. infestans* MP 946-potato interaction. Importantly, the nitroproteome profiles varied for the pathogen virulence pattern and comparative analysis revealed that *vr* MP 977 *P. infestans* represented a much more diverse quality spectrum of nitrated proteins. Abundance profiles of nitrated proteins that were up- or downregulated were substantially different also between the analyzed growth phases. Briefly, *in planta* growth of *avr* and *vr P. infestans* was accompanied by exclusive nitration of proteins involved in energy metabolism, signal transduction and pathogenesis. Importantly, the *P. infestans-*potato interaction indicated cytosolic RXLRs and Crinklers effectors as potential sensors of RNS. Taken together, we explored the first plant pathogen nitroproteome. The results present new insights into RNS metabolism in *P. infestans* indicating protein nitration as an integral part of pathogen biology, dynamically modified during its offensive strategy. Thus, the nitroproteome should be considered as a flexible element of the oomycete developmental and adaptive mechanism to different micro-environments, including host cells.

## Introduction

Nitric oxide (NO) is a crucial signaling molecule during both the highly conserved pathogen-associated molecular pattern (PAMP) triggered immunity (PTI) and in a highly specific effector-triggered immunity (ETI) (e.g., Asai and Yoshioka, 2009; Perchepied et al., 2010; Bellin et al., 2013; Schlicht and Kombrink, 2013). Although NO is neither a strong oxidant nor a strong reductant (Bartesaghi and Radi, 2018), the NO burst occurring at the site of attempted host colonization may effectively stimulate a further sequence of defense events (Arasimowicz-Jelonek and Floryszak-Wieczorek, 2011; Vandelle and Delledonne, 2011). It is well established that the signaling mode of NO action at the molecular level involves post-translational modification (PTM) of proteins including protein tyrosine (Tyr) residue nitration *via* the NO-derived molecule - peroxynitrite (ONOO^-^). Although this reactive nitrogen species (RNS) and ONOO^-^-dependent protein nitration were first identified in animals as toxic events contributing to oxidative and nitrosative stress in the biological milieu, there is increasing evidence that Tyr nitration is a selective process with a well-defined target (Radi, 2013). According to Bartesaghi et al. (2007) nitro-Tyr yield is low under physiological conditions and only 1–5 nitro-Tyr residues per 10,000 tyrosines is detectable. It has also been shown that the phenomenon is dependent on the protein composition, structure, its intracellular concentration, localization and interaction with other molecules (Abello et al., 2009). This PTM affects the activity, stability or the intracellular location of proteins, thus potentially altering their functions and signal transduction (Greenacre and Ischiropoulos, 2001). It has been experimentally confirmed that nitration of critical Tyr residues may lead to both activating and suppressing of protein function (e.g., Ji et al., 2006; Chaki et al., 2009b; Begara-Morales et al., 2015; Yeo et al., 2015). Interestingly, some data showed that the selective nitration of Tyr residues *via* ONOO^-^ may also interfere with signaling processes associated with protein Tyr phosphorylation (Monteiro et al., 2008; Aburima et al., 2010).

Importantly, generation and potential functions of NO during biotic interactions have so far been analyzed solely from the point of view of the host plant. However, too often the role of NO within the pathogen is ignored when considering plant-pathogen interactions (Mur et al., 2006; Arasimowicz-Jelonek and Floryszak-Wieczorek, 2014, 2016). Recent advances in understanding of plant-pathogen systems have shown that plant pathogens are also capable of NO synthesis and might employ NO to colonize the host (Turrion-Gomez and Benito, 2011; Samalova et al., 2013). Unfortunately, the functional role of RNS in this systematically heterogeneous group of microorganisms has not been thoroughly studied to date. However, eukaryotic plant pathogens belonging to *Fungi* and fungus-like *Oomycetes*, potentially cope with various NO biosynthetic and NO detoxification routes (Arasimowicz-Jelonek and Floryszak-Wieczorek, 2016). For example, NO generation has been detected in mycelia of *Blumeria graminis* (Prats et al., 2008), *Oidium neolycopersici* (Piterková et al., 2011), *Magnaporthe oryzae* (Samalova et al., 2013) and in hyphae and infection structures of the oomycete *Bremia lactucae* (Sedlárová et al., 2011). Although, precise mechanism of NO-mediated regulation in plant pathogens has not been yet clarified, experimental data revealed NO participation during pathogen development, virulence and its survival in the host (Arasimowicz-Jelonek and Floryszak-Wieczorek, 2014, 2016). Since protein nitration is a universal mechanism of intracellular signaling found in organisms belonging to distinct systematic groups, it may be suspected that similarly, to mammals and plants Tyr nitration might greatly affect protein function also in filamentous plant pathogens, such as e.g., fungi and oomycetes. In confirmation, the first and to date the only report revealed the presence of nitrated proteins in the crude extract of the sunflower downy mildew oomycete *Plasmopara halstedii*, however, the identification and potential relevance of this PTM in the pathogen biology remains unexplored (Chaki et al., 2009a).

The oomycete *Phytophthora infestans* (Mont.) de Bary is a causative agent of late blight disease responsible for the great Irish famine in the mid-nineteenth century. Currently, late blight is considered as the most devastating disease of potato (*Solanum tuberosum* L.), generating costs of up to one billion euro per year in the EU alone (Resjö et al., 2017). In turn, potato is the largest non-cereal food crop worldwide and the fourth most important food crop after rice, wheat and maize with 300 million tons of annual global production (Raymundo et al., 2018). Thus, the pathogen is regarded as a threat to global food security. Although *P. infestans* has been studied for more than a century, progress made on disease control of host crops is still unsatisfactory, in part due to the fast evolution and adaptive capacity of the pathogen to new micro-environments (Forbes, 2012).

Since environmental adaptation of pathogens could be achieved at the molecular level through PTM of proteins, knowledge of NO-dependent PTM in biology and pathobiology of hemibiotrophic *P. infestans* seems to be essential. In the present study we characterized the NO metabolic status defined as the nitroproteome pattern during *in vivo* and *in planta P. infestans* hyphal growth conditions. An experimental approach involved avirulent (*avr*) and virulent (*vr*) *P. infestans* isolates (in reference to the potato genotype ‘Sarpo Mira’), creating a useful background for the identification of candidates of protein nitration, favorable host colonization.

## Materials and Methods

### Pathogen Culture

*Phytophthora infestans* (Mont.) de Bary – the avirulent isolate MP 946 (race 1.3.4.7.10.11) and the virulent MP 977 (race 1.2.3.4.6.7.10) in reference to the potato cv. Sarpo Mira, was provided by the Plant Breeding Acclimatization Institute (IHAR), Research Division in Młochów, Poland. For *in vitro* studies: the oomycete was grown for 9 days in the dark on a cereal-potato medium supplemented with dextrose. For *in planta* studies: potato plants were inoculated by spraying with 3 ml of a freshly prepared suspension of sporangia and zoospores (5.0 × 10^5^ sporangia per ml) and incubated in a sterile boxes for 9 days at 16°C and 95% relative humidity in the darkness (sporangia of *P. infestans* were obtained by washing 14-day-old cultures with cold distilled water and zoospores were released by incubating the sporangia in water at 4°C for 2 h). The area under disease progress was evaluated on potato leaves 9 days after inoculation using a scale from I to IV (James, 1971), which represented the percentage of leaf area covered by late blight symptoms (I = 1 to 9%; II = 10 to 24%; III = 25 to 49%; IV = 50 to 100%; Supplementary Figure S1).

The hyphae growing *in vitro* or *in planta* were manually collected, frozen in liquid nitrogen and stored at -80°C. Additionally, hyphae *in planta* were isolated by dipping the infected leaf in 5% cellulose acetate (dissolved in acetone), letting the acetone evaporate, and stripping the cellulose acetate film off the leaves according to Both et al. (2005).

### Plant Material

Plants of the tetraploid potato cv. Sarpo Mira came from the Potato Gene bank (Plant Breeding and Acclimatization Institute – IHAR-PIB in Bonin, Poland). Potato seedlings propagated through *in vitro* culture were transferred to the soil (a composition of high peat moss approx. 50% in the substrate) and kept in a phytochamber with 16 h of light (180 μmol⋅m^-2^⋅s^-1^) at 18 ± 2°C and 60% humidity up to the stage of ten leaves.

### Nitric Oxide and Peroxynitrite Detection

Nitric oxide and peroxynitrite formation was measured quantitatively using Cu-FL (2-{2-chloro-6-hydroxy-5-[2-methylquinolin-8-ylamino)methyl]-3-oxo-3H-xanthen-9-y1}benzoic acid, Strem Chemicals) and APF (aminophenyl fluorescein, Sigma), respectively. Pathogen samples (0.1 g) were incubated in the dark for 1 h in a mixture containing 10 μM Cu-FL in 10 mM Tris–HCl buffer (pH 7.2) or 5 μM APF in 100 mM phosphate buffer (pH 7.4). After the incubation, the probes were transferred into 24-well plates (1 ml per well). Fluorescence in the reaction was measured using spectrofluorometer (Fluorescence Spectrometer Perkin Elmer LS50B, United Kingdom) at 488 nm excitation and 516 nm emission (for NO detection) and 490 nm excitation and 510 nm emission (for ONOO^-^ detection) filters. Fluorescence was expressed as relative fluorescence units. Additionally, 1 mM cPTIO or 50 μM ebselen were used to scavenge NO and ONOO^-^, respectively.

### Reactive Nitrogen Species Detection by Fluorescence Microscopy

Nitric oxide formation was detected using a fluorescent DAF-2DA dye (Calbiochem), while ONOO^-^ production was localized using the fluorescent reagent aminophenyl fluorescein, APF (Sigma). *Phytophthora infestans* isolates were incubated with 10 mM DAF-2DA or 10 mM APF, prepared in 10 mM Tris–HCl (pH 7.4) for 1 h at room temperature. Additionally, 1 mM cPTIO or 50 μM ebselen were used to scavenge NO and ONOO^-^, respectively. After incubation, samples were washed several times in the same buffer and immediately examined under a Zeiss LSM 510 confocal microscope (Carl Zeiss, Jena, Germany) using standard filters and collection modalities for DAF-2DA (excitation 495 nm; emission 515 nm) and APF (excitation 490 nm; emission 515 nm) green fluorescence. Images were processed and analyzed using an LSM Image Browser (Zeiss).

### Peroxynitrite Generator and Scavenger Treatment

To evaluate the effect of exogenous ONOO^-^, *P. infestans* hyphae were sprayed with ONOO^-^ generator (50 μM 3-Morpholinosydnonimine, SIN-1, Calbiochem), which gradually decomposed to yield equimolar amounts of NO and O_2_^-^. Scavenger of ONOO^-^ (50 μM ebselen, Sigma) were used to estimate the effect of endogenous ONOO^-^ on protein Tyr nitration. Control cultures were treated with sterile water. After 24 h of hyphae incubation protein 3-nitrotyrosine assay was performed.

### Protein 3-Nitrotyrosine Assay

Pathogen samples (0.2 g) were ground in liquid nitrogen to a fine powder, suspended in 50 mM Tris–HCl buffer (pH 7.6) with 2 mM EDTA, 2 mM DTT and 1 mM PMSF and centrifuged at 10 000 *g* for 15 min at 4°C. The protein concentration in the supernatant was determined with the Bradford (1976) assay, using BSA as the standard. 3-nitrotyrosine in a protein sample was measured using the OxiSelect^TM^ Nitrotyrosine ELISA Kit (Cell Biolabs Inc.) according to the manufacturer’s instruction.

### Protein Extraction, 2D Gel Electrophoresis and Western Blotting

Protein extraction for 2D electrophoresis was carried out according to Méchin et al. (2007). Briefly, pathogen samples (0.5 g) were ground in liquid nitrogen to a fine powder and proteins were precipitated with a cold TCA-acetone solution with the addition of 2-mercaptoethanol (0.07%) for minimum 1 h at -20°C. The proteins were then solubilized using DeStreak Rehydration Solution (GE HealthCare) supplemented with 20 mM DTT and 0.2% Bio-Lyte buffer (Bio-Rad). The concentration of proteins in the final samples was evaluated with the use of a commercial 2-D Quant Kit (GE Healthcare), according to the manufacturer’s instruction. Approximately 100 μg of proteins were loaded onto 7 cm IPG strips with 3–10 pH gradients (Bio-Rad). The strips were rehydrated overnight and used for isoelectrofocusing (IEF) using a Protean IEF cell system (Bio-Rad). The run was carried out in the following order: (i) 300 V (1 h), (ii) 3500 V (1.5 h), and (iii) 3500 V (total 20,000 Vh). For the SDS-PAGE, the strips were equilibrated 2 times for 15 min in an equilibration buffer (50 mM Tris–HCl, pH 8.8, 6 M urea, 30% glycerol, 2% SDS, 0.002% Bromophenol blue), first containing 65 mM DTT, followed by an equilibration buffer with 135 mM iodoacetamide. For separation in the second dimension the strips were applied to 10% precast polyacrylamide gels (Bio-Rad) and run in a Mini-PROTEAN Tetra Cell (Bio-Rad) at a constant current (20 mA per gel) with a Prestained Protein Ladder (Thermo Scientific). For Western blot analyses, proteins were transferred to PVDF membranes. After transfer, membranes were used for cross-reactivity assays with rabbit polyclonal antibodies against nitrotyrosine (Life Technologies, 1:1000 dilutions). To check the specificity of the anti-nitrotyrosine antibodies, sodium dithionite solution was used to reduce nitrotyrosine to aminotyrosine (Supplementary Figure S2). Briefly, the control PVDF membrane was incubated with solution of 10 mM sodium dithionite in 50 mM pyridine-acetate buffer (pH 5.0) for 1 h at room temperature. After the reaction, the membrane was equilibrated with 0.2% v/v Tween-20 in Tris–buffered saline and then incubated with anti-nitrotyrosine antibody as described above. For immunodetection the goat anti-rabbit antibody conjugated to horseradish peroxidase (Agrisera) and Lumi-Light western blotting substrate (Roche) was used. At least three independent blots from different experiments were analyzed. The intensity of spots was quantified using a ChemiDoc MP Imaging System (Bio-Rad) coupled with a high-resolution camera, and protein spot intensity was analyzed by means of the MultiGauge (release 2.2) Fuji software. The quantitative results of protein Tyr nitration were calculated using MultiGauge software (Fuji) by summing the pixels intensity within each protein spot image, and the data were presented in comparison to the control sample average of 0 (control – *in vitro* growth of *P. infestans*).

### Mass Spectrometry (MS) and Protein Identification

Protein identification was performed using liquid chromatography coupled to the mass spectrometer at the Laboratory of Mass Spectrometry, Institute of Biochemistry and Biophysics, Polish Academy of Sciences (Warsaw, Poland). Protein spots were carefully cut out from the PVDF membranes manually under a binocular microscope using a sterile, disposable razor blade and subjected to the standard procedure of trypsin digestion as described earlier (Arasimowicz-Jelonek et al., 2016). Briefly, a concentrated and desalted peptide solution was separated on a nano-Ultra Performance Liquid Chromatography RP-C18 column (Waters, BEH130 C18 column, 75 mm i.d., 250 mm long) of a nanoACQUITY UPLC system, using a 45 min linear acetonitrile gradient. Column outlet was directly coupled to the electrospray ionization (ESI) ion source of the Orbitrap Velos type mass spectrometer (Thermo), working in the regime of data dependent MS to MS/MS switch with the HCD type peptide fragmentation. An electrospray voltage of 1.5 kV was used. Raw data were pre-processed with the Mascot Distiller software (v. 2.4.2.0; Matrix Science), then obtained peptide masses and fragmentation spectra were matched to the *Phytophthora* UniProt database (18,581 sequences; 7,696,749 residues) using the Mascot search engine (Mascot Daemon v. 2.4.0, Mascot Server v. 2.4.1, and Matrix Science). The following parameters were adopted for database searches: enzyme specificity was set to semiTrypsin, peptide mass tolerance to 20 ppm and fragment mass tolerance to 0.1 Da. The protein mass was left as unrestricted, and mass values as monoisotopic with one missed cleavages being allowed. Alkylation of cysteine by carbamidomethylation was fixed and oxidation of methionine and carboxymethylation on lysine were set as a variable modification. Protein identification was performed using the Mascot search engine, with the probability-based algorithm. The expected value threshold of 0.05 was used for analysis, which means that all peptide identifications had less than a 1 in 20 chance of being a random match.

### Statistical Analysis

All results are based on three biological replicates derived from three independent experiments. For each experiment, means of the obtained values (*n* = 9) were calculated along with standard deviations. To estimate the statistical significance between means, the data were analyzed with the use of one-way analysis of variance (ANOVA) followed by a Dunnett’s test at the level of significance α = 0.05 or α = 0.01. The differences between protein spots in both *P. infestans* growth phases were determined using ANOVA followed by a student *t*-test with *p* < 0.05 as the limit of significance.

## Results and Discussion

### Reactive Nitrogen Species Generation in *P. infestans* Structures

**FIGURE 1:**
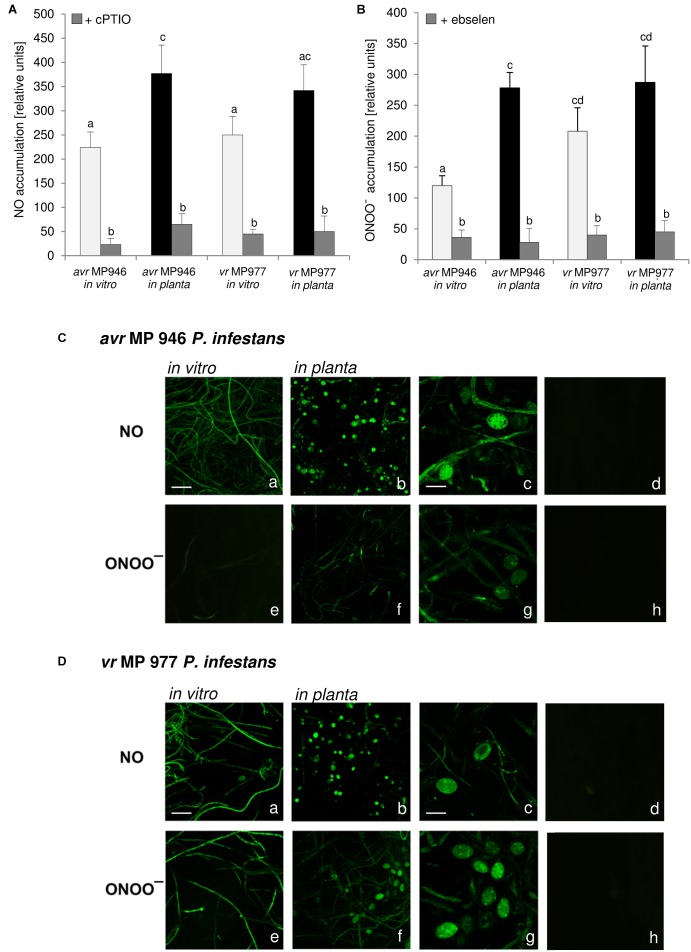
Reactive nitrogen species formation in *P. infestans* growing under *in vitro* and *in planta* conditions. **(A)** Nitric oxide and **(B)** peroxynitrite accumulation measured as Cu-Fl and APF fluorescence, respectively. Values represent the mean ± SD of three biological replicates derived from three independent experiments (*n* = 9). Columns marked with the same letter are not significantly different (Dunnett’s test) at *p* < 0.05. Bio-imaging of nitric oxide and peroxynitrite formation in **(C)** *avr* MP 946 and **(D)** *vr* MP 977 *P. infestans*. Pathogen structures growing *in vitro* **(a,e)**, *in planta* **(b–d, f–h)** and *in planta* in the presence of 1 mM cPTIO **(d)** or 50 μM ebselen **(h)**. Images show general phenomena representative of three individual experiments. Bars indicate 20 μm **(a,b,e,f)** and 100 μm **(c,d,g,h)**.

Nitric oxide and ONOO^-^ production in *P. infestans* growing *in vitro* and *in planta* were detected quantitatively using DAF-2DA and APF fluorochrome, respectively. The experiment documented that both isolates, differing in their virulence pattern on the ‘Sarpo Mira’, are able to generate RNS (Figure 1). However, *vr* MP 977 presented significantly higher levels of ONOO^-^ under *in vitro* conditions (Figure 1A,B). The interaction with the plant led to boosted ONOO^-^ formation mainly in the a*vr* MP 946 isolate (Figure 1B). Additional real-time imaging of RNS production in both *P. infestans* isolates revealed green fluorescence localized particularly in hyphae and mature sporangia growing on the medium and *in planta*, respectively (Figure 1C,D). Importantly, a*vr* MP 946 *P. infestans* presented far weaker ONOO^-^ formation when growing on the medium and only contact with potato plants provoked a considerable increase in ONOO^-^ emission (Figure 1B,C).

As stated earlier, pathogens belonging to *Fungi* and fungus-like *Oomycetes* have active sources of NO and pathways of its detoxication, which defend them against NO-induced damages and ensure the vital level required for the signaling function both in the pathogen physiological state and during host tissue colonization (Arasimowicz-Jelonek and Floryszak-Wieczorek, 2014). Importantly, RNS production observed in *P. infestans* was accelerated during *in planta* sporangia development. It can be assumed that NO and ONOO^-^ could mediate nuclear division, or degeneration of a proportion of the nuclei in the sporangium providing sporangia with the ability to release zoospores in rapid succession. The presence of NO was also observed in the infection structures of another oomycete, *B. lactucae*, grown both on susceptible and resistant lettuce cultivars, however, the plant genotype determined the timing of the pathogen development. A strong NO signal was detected in the tip of the germ tube and the appressorium, which is a prerequisite for tissue penetration. A weaker NO signal was detected in developing primary and secondary vesicles, intracellular hyphae and in haustoria on susceptible lettuce (Sedlárová et al., 2011).

### Peroxynitrite Formation in *P. infestans* Is Accompanied by Protein Tyrosine Nitration

**FIGURE 2:**
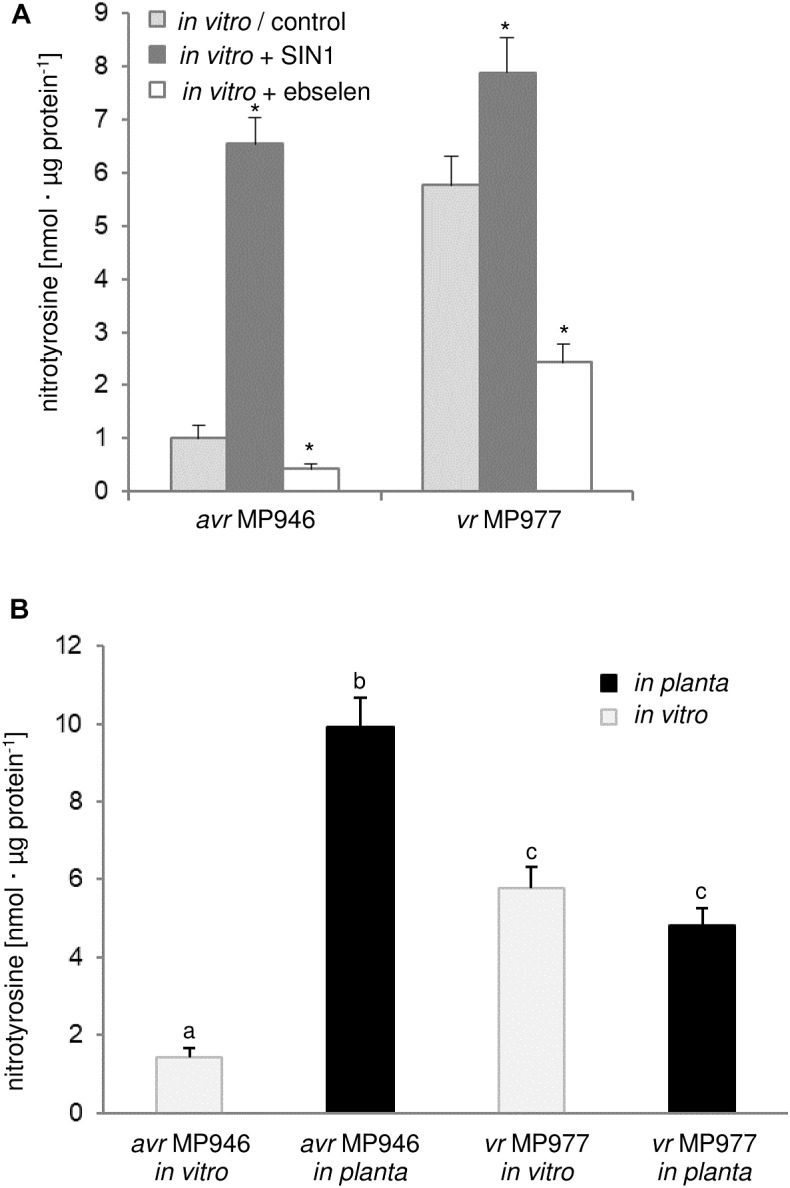
Quantification of protein Tyr nitration of *avr* MP 946 and *vr* MP 977 *P. infestans*. **(A)** Quantification of nitrated proteins measured as 3-nitrotyrosine content in *avr* MP 946 and *vr* MP 977 *P. infestans* structures enriched or depleted with ONOO^-^ during *in vitro* growth; 3-nitrotyrosine content was estimated 5 h after *P. infestans* pretreatment with 50 μM SIN-1 or 50 μM ebselen; asterisks indicate values that differ significantly from the control (untreated) culture of *avr* MP 946 or *vr* MP 977 at ^∗^*p* < 0.05. **(B)** 3-nitrotyrosine content in *avr* MP 946 and *vr* MP 977 *P. infestans* growing under *in vitro* and *in planta* conditions; the results were calculated using the standard curve and expressed as nmol 3-nitrotyrosine/μg protein; values represent the mean ± SD of three biological replicates derived from three independent experiments (*n* = 9); columns marked with the same letter are not significantly different (Dunnett’s test) at *p* < 0.05.

There is evidence that ONOO^-^ fulfills an important role during the plant-pathogen interaction creating a cellular redox milieu toward plant defense expression (Durner et al., 1998; Alamillo and García-Olmedo, 2001; Arasimowicz-Jelonek et al., 2016). However, there is no information available on the ONOO^-^ mode of action in relation to any fungal and fungal-like pathogens. Searching for the functional role of the RNS in *P. infestans* we identified a protein pool potentially targeted by ONOO^-^ *via* nitration. Firstly, we analyzed the abundance of nitrated proteins in *P. infestans* hyphae of *avr* MP 946 and *vr* MP 977 isolates during *in vitro* and *in planta* conditions (Figure 2). Based on immunoassay we found that the total amount of nitrated proteins varied greatly between isolates during both *in vitro* and *in planta* growth phases. A considerable increase in ONOO^-^ formation noted in the *avr* MP 946 isolate during contact with the host tissues was accompanied by the highest level of the total protein pool undergoing nitration. It was *ca*. 9-fold greater in comparison to *in vitro* growth conditions of *avr* MP 946. In contrast, quantitative changes in the nitrated protein pool of *vr* MP 977 *P. infestans* growing *in planta* revealed only a slightly lowered expression of nitrated proteins (Figure 2B).

As we indicated before, potato ETI is accompanied by a periodic *ca.* 3-fold induction of ONOO^-^ production starting within the first hour after challenge with the oomycete (Arasimowicz-Jelonek et al., 2016). Thus, *avr* MP 946 *P. infestans* during the contact with potato was exposed to both innate ONOO^-^ and host-derived ONOO^-^ (Arasimowicz-Jelonek et al., 2016; Izbiańska et al., 2018). In consequence, boosted and pathophysiological levels of RNS *in planta* may result in an elevated level of nitrated proteins within pathogen structures. Moreover, *avr* MP 946 features a relatively low-efficient strategy to remove RNS and suppress its excessive accumulation (data not presented), which additionally accelerates *in planta* homeostasis misbalance and promotes pathogen subjugation. In contrast, *vr* MP 977 seems to be perfectly adapted to both plant- and pathogen-derived RNS production, since only slight fluctuation in the total protein pool undergoing nitration was observed during late blight development. Therefore, changes in the total protein pool undergoing Tyr nitration phenomena in *P. infestans* structures in response to the host may reflect homeostasis misbalance connected to stress-associated conditions. It is generally accepted that RNS depending on its abundance within the cellular milieu may provoke opposite effects (Yu et al., 2014). An elevated level of ONOO^-^ contributes to oxidative and nitrosative stress in cells and Tyr nitration has been assumed to be a reliable marker of nitro-oxidative stress, since this PTM is frequently associated with pathophysiological states (Corpas et al., 2007). On the other hand, nitrated proteins may also be present in living cells under optimal physiological conditions, which strengthens the hypothesis that *via* Tyr nitration ONOO^-^ could be involved in cooperation with NO in a broad spectrum of signaling and regulatory processes (Arasimowicz-Jelonek and Floryszak-Wieczorek, 2011; Vandelle and Delledonne, 2011). It should be noted that pathogens may employ nitration phenomenon to cause disease. For example, *Streptomyces* spp. including *S. scabies, S. acidiscabies* and *S. turgidiscabies* produce phytotoxin thaxtomin, a nitrated dipeptide which inhibits cellulose synthesis (Bignell et al., 2014). Moreover, nitration of lipopeptide arylomycin produced by *Streptomyces* sp. Tü 6075 improves its antibacterial activity (Schimana et al., 2002).

### Nitroproteome-Wide Identification of Tyrosine Nitration in *P. infestans*

**FIGURE 3:**
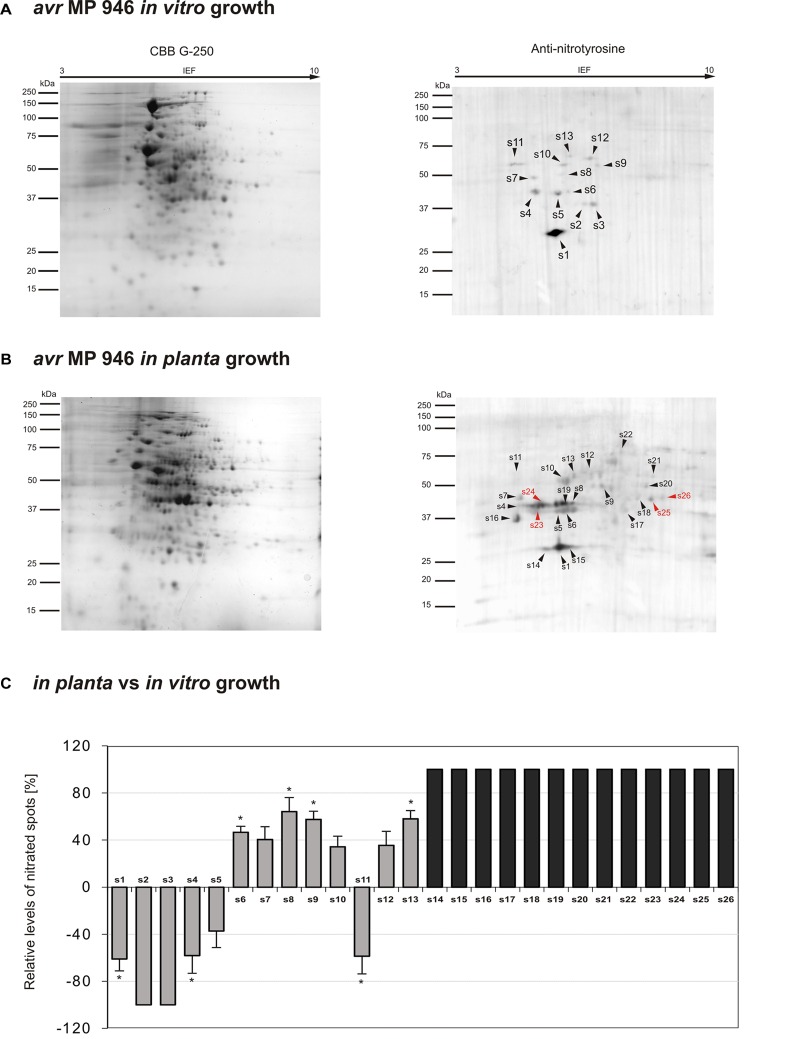
Tyrosine nitration pattern of *avr* MP 946 *P. infestans* growing **(A)** *in vitro* and **(B)** *in planta*. Representative 2D electrophoresis (pH 3–10 for the first dimension) of *avr P. infestans* stained with CBB G-250 and representative immunoblots probed with a polyclonal antibody against nitrotyrosine diluted at 1:1000. Molecular-mass standards (kDa) are indicated on the left. Arrowheads indicate all the immunoreactive spots, the symbols (s1–s26) refer to the proteins listed in Table 1. Red arrowheads indicate spots specific to the *avr* MP 946 *P. infestans* growing phase. **(C)** The quantitative results of protein Tyr nitration were calculated by summing the pixels intensity within each protein spot image, and the data were presented in comparison to the control sample average of 0 (control – *in vitro* growth phase of *avr* MP 946 *P. infestans*). Asterisks indicate values that differ significantly from the control at ^∗^*p* < 0.05.

**FIGURE 4:**
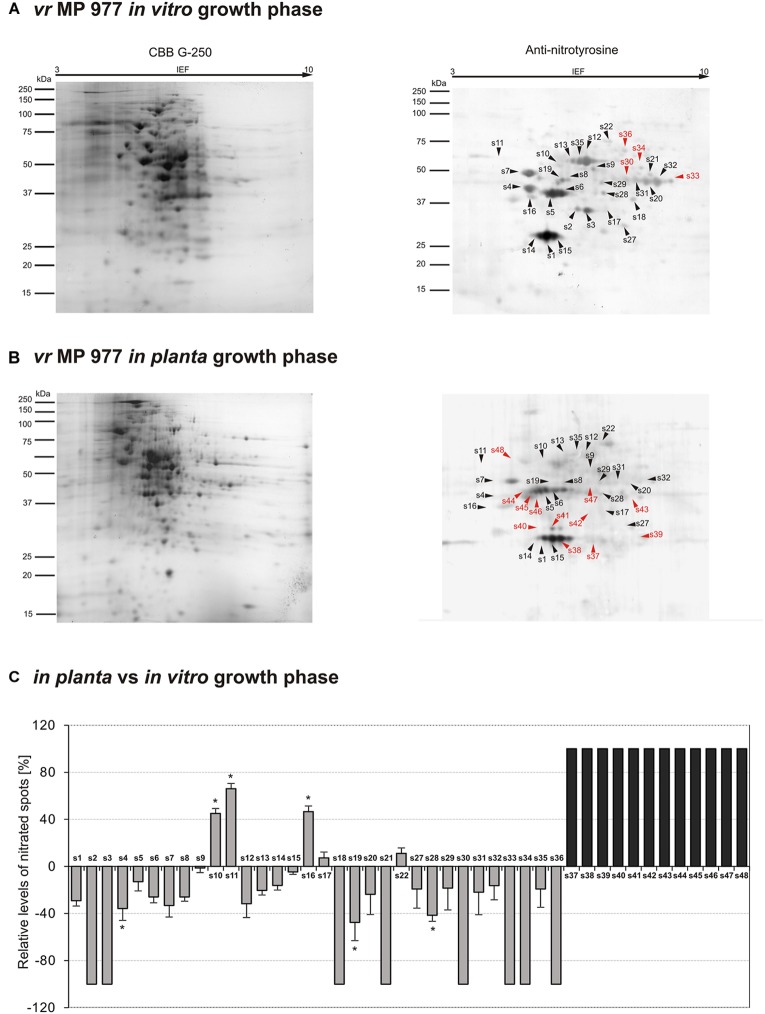
Tyrosine nitration pattern of *vr* MP 977 *P. infestans* growing **(A)** *in vitro* and **(B)** *in planta*. Representative 2D electrophoresis (pH 3–10 for the first dimension) of *avr P. infestans* stained with CBB G-250 and representative immunoblots probed with a polyclonal antibody against nitrotyrosine diluted at 1:1000. Molecular-mass standards (kDa) are indicated on the left. Arrowheads indicate all the immunoreactive spots, the symbols (s1–s48) refer to the proteins listed in Table 1. Red arrowheads indicate spots specific to the *vr* MP 977 *P. infestans* growing phase. **(C)** The quantitative results of protein Tyr nitration were calculated by summing the pixels intensity within each protein spot image, and the data were presented in comparison to the control sample average of 0 (control – *in vitro* growth phase of *vr* MP 977 *P. infestans*). Asterisks indicate values that differ significantly from the control at ^∗^*p* < 0.05.

The observed quantitative differences have encouraged us to take the next step aimed at nitrated protein identification. Identification of 3-nitrotyrosine-containing proteins and mapping of nitrated residues is a challenging task due to the limited incidence of this modification in biological samples (Abello et al., 2009). Importantly, a low number of proteins are preferential targets of nitration (usually fewer than 100 proteins per proteome), in contrast with the large number of proteins modified by other post-translational events such as phosphorylation, acetylation and, notably, S-nitrosylation (Batthyány et al., 2017). In our study, Tyr nitration protein patterns of *P. infestans* isolates during *in vitro* and *in planta* growth were detected with an antibody against nitrotyrosine (Figure 3, 4). A total of 48 spots detected in *avr* MP 946 and *vr* MP 977 isolates during all three immuno-blot biological replicates were analyzed by LC–MS–MS/MS mass spectrometry after trypsin digestion using the MASCOT search engine to analyze MS data in order to identify proteins from primary-sequence databases. The identified immunopositive proteins are listed in Table 1. In some cases the selected proteins derived from *P. infestans* were identified as heterogeneous with more than one protein identified within them, however, a majority of spots were homogenous, containing one specific protein and clear identifications was demonstrated (Table 1). However, these proteins should be considered putatively nitrated until the nitration sites have been identified by sequence analysis.

**Table 1:** Nitrated proteins in *P. infestans* growing *in vitro* and *in planta* isolated from PVDF membrane and identified by LC-MS-MS/MS.

No.	Protein name	Score	Gene ID	Functional category
S1	Putative uncharacterized protein	105	PITG_12377	Nucleobase-containing compound metabolism
S2	Histone H4^∗^	110	PITG_03552	Nucleobase-containing compound metabolism
S3	Tubulin alpha chain^∗^	94	PITG_07996	Cytoskeleton organization
S4	Heat shock protein 90^∗^ 14-3-3 protein epsilon Calmodulin^∗^	138 137 91	PITG_06415 PITG_19017 PITG_06514	Cell redox homeostasis / response to stress Signal transduction Signal transduction
S5	Fructose-bisphosphate aldolase^∗^ Histone H3^∗^ Peroxiredoxin-2^∗^	76 60 56	PITG_02786 PITG_03551 PITG_15492	Carbohydrate metabolism / energy metabolism Nucleobase-containing compound metabolism Cell redox homeostasis / response to stress
S6	PREDICTED: ATP-dependent RNA helicase SUPV3L1	122	PITG_11795	Nucleobase-containing compound metabolism
S7	Putative uncharacterized protein	72	PITG_00319	Proteolysis / protein metabolism
S8	PREDICTED: Structural maintenance of chromosomes protein	60	PITG_05757	Nucleobase-containing compound metabolism
S9	Putative uncharacterized protein	137	PITG_12490	Signal transduction
S10	NADH dehydrogenase [ubiquinone] flavoprotein 1, mitochondrial	60	PITG_06815	Pathogenesis
S11	Glyceraldehyde-3-phosphate dehydrogenase^∗^	95	PITG_01938	Carbohydrate metabolism / energy metabolism
S12	Putative uncharacterized protein Actin-like protein	152 78	PITG_06853 PITG_15078	Endonuclease activity / protein metabolism Cytoskeleton organization
S13	Xanthine dehydrogenase	58	PITG_02284	Oxidation-reduction process / others
S14	PREDICTED: Secreted RxLR effector peptide protein, (fragment) Secreted RxLR effector peptide protein PREDICTED: Secreted RxLR effector peptide protein	25 20 19	PITG_05074 PITG_23137 PITG_15764	Signal transduction / pathogenesis Signal transduction / pathogenesis Signal transduction / pathogenesis
S15	Triosephosphate isomerase^∗^	144	PITG_13116	Carbohydrate metabolism / energy metabolism
S16	60S acidic ribosomal protein P0	728	PITG_17261	Ribosome biogenesis / protein metabolism
S17	Guanine nucleotide-binding protein subunit beta-2-like protein	343	PITG_09556	Ribosome biogenesis /signal transduction
S18	Aspartate aminotransferase	574	PITG_02256	Carbohydrate metabolism / energy metabolism
S19	PREDICTED: NmrA-like family protein	216	PITG_14492	Cell redox homeostasis / response to stress
S20	Glutamate dehydrogenase^∗^	194	PITG_07671	Nitrogen metabolism / protein metabolism
S21	Putative uncharacterized protein	143	PITG_13946	Signal transduction
S22	Phosphoenolpyruvate carboxykinase	1178	PITG_10210	Carbohydrate metabolism / energy metabolism
S23	PREDICTED: Secreted RxLR effector peptide protein	24	PITG_06059	Hydrolase activity / pathogenesis
S24	PREDICTED: Alcohol dehydrogenase	103	PITG_10292	Oxidoreductase activity / others
S25	PREDICTED: Type I inositol-3,4-bisphosphate 4-phosphatase	48	PITG_12548	Signal transduction
S26	Peroxiredoxin-like protein^∗^	123	PITG_00585	Cell redox homeostasis / response to stress
S27	Carbonic anhydrase^∗^	292	PITG_00682	Carbohydrate metabolism / energy
S28	PREDICTED: Secreted RxLR effector peptide protein	23	PITG_04081	Signal transduction / pathogenesis
S29	PREDICTED: Voltage-gated potassium channel subunit beta	184	PITG_03719	Transport / signal transduction
S30	PREDICTED: Secreted RxLR effector peptide protein	22	PITG_01904	Signal transduction / pathogenesis
S31	Transient receptor potential Ca2 channel (TRP-CC) family protein	86	PITG_05737	Signal transduction
S32	Heat shock 70 kDa protein Histone H2B	205 114	PITG_11244 PITG_03550	Cell redox homeostasis / response to stress Nucleobase-containing compound metabolism
S33	Putative uncharacterized protein	74	PITG_16013	Unclassified
S34	PREDICTED: NAD-specific glutamate dehydrogenase	71	PITG_08802	Nitrogen metabolism / protein metabolism
S35	PREDICTED: Inositol-3-phosphate synthase Pyruvate kinase^∗^	404 91	PITG_01700 PITG_09400	Lipid metabolism / energy metabolism Carbohydrate metabolism / energy metabolism
S36	3-ketoacyl-CoA thiolase, mitochondrial	187	PITG_07197	Lipid metabolism / energy metabolism
S37	Crinkler (CRN) family protein	28	PITG_23273	Host-translocated effectors / pathogenesis
S38	Triosephosphate isomerase^∗^	122	PITG_16048	Carbohydrate metabolism / energy metabolism
S39	Putative uncharacterized protein	77	PITG_12486	tRNA processing / nucleobase-containing compound metabolism
S40	Putative uncharacterized protein	78	PITG_00160	Signal transduction
S41	Crinkler (CRN) family protein	24	PITG_20172	Host-translocated effectors / pathogenesis
S42	Prohibitin	210	PITG_00827	DNA replication / nucleobase-containing compound metabolism
S43	Putative uncharacterized protein	114	PITG_01555	Cytoskeleton organization
S44	Serine/threonine protein kinase	99	PITG_14221	Protein phosphorylation / signal transduction
S45	Syntaxin-like protein	42	PITG_01470	Intracellular protein transport / signal transduction
S46	PREDICTED: Choline/Carnitine O-acyltransferase	42	PITG_17907	Transferase activity / other
S47	2-amino-3-ketobutyrate coenzyme A ligase, mitochondrial	89	PITG_03685	Oxidation-reduction process / other
S48	ATP synthase subunit beta	533	PITG_06595	ATP metabolic process / energy metabolism


*Asterisks indicate proteins which have previously been functionally confirmed as nitrated in animal or plant systems.*

**FIGURE 5:**
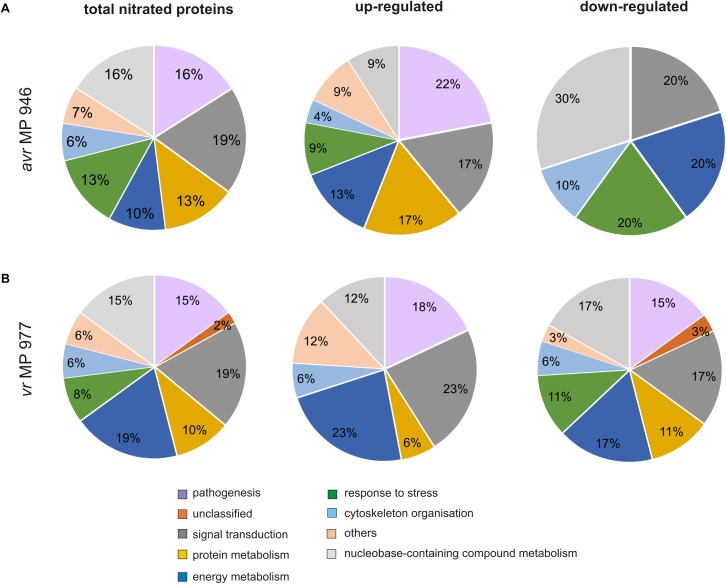
Functional classification and distribution of 48 nitrated protein spots identified by LC–MS–MS/MS analysis in **(A)** *avr* MP 946 and **(B)** *vr* MP 977 *P. infestans*. The area for each group indicates the relative percentage of proteins (%) in that group. The color code represents the functional classification according to the UniProt database.

To gain further insights into the nitroproteome of *P. infestans*, the proteins identified from both pathogen isolates were classified into nine functional categories corresponding to different biological processes, which are displayed in Figure 5. The category containing the highest number of identified protein candidates for nitration in both isolates is related to signal transduction (19%), followed by proteins related to metabolic process requiring nucleobase containing compounds (15–16%) and pathogenesis (15–16%). Importantly, in *vr* MP 977, the proteins associated with energy metabolism constituted the most-represented functional category in addition to proteins involved in signal transduction (19%) (Figure 5B). These results indicate that the nitrated proteins, widely distributed within pathogen structures, are involved in a variety of processes in *P. infestans*.

Importantly, 12 of the identified proteins have previously been functionally confirmed to be nitrated in animal or plant systems (Table 1). These include histones H3, H4 and pyruvate kinase (Ghesquière et al., 2009), tubulin alpha chain (Tedeschi et al., 2005), heat shock protein 90 (Franco et al., 2013), calmodulin (Smallwood et al., 2003), peroxiredoxin-2 (Randall et al., 2014, 2016), glyceraldehyde-3-phosphate dehydrogenase (Palamalai and Miyagi, 2010), fructose 1,6-bisphosphatase (Koeck et al., 2004), glutamate dehydrogenase (Piszkiewicz et al., 1971), carbonic anhydrase (Chaki et al., 2013) and triosephosphate isomerase (Gao et al., 2017).

### *P. infestans* Virulence Pattern Determine Nitroproteome Status

Proteomic analysis of nitrated proteins in hyphae of *avr* MP 946 *P. infestans* resulted in the identification of 13 spots during *in vitro* (Figure 3A) and 24 spots during *in planta* phases, respectively (Figure 3B). Eleven protein spots were found to be common for both growth conditions; in turn, as many as 2 (s2, s3) spots were specific to *in vitro* growth and 13 (s14–s26) spots were specific to *in planta* growth (Figure 3C in gray color). Importantly, 4 out of 13 spots appearing *in planta* were exclusively nitrated in hyphae of *avr* MP 946 *P. infestans* growing on potato (s23–s26, Figure 3B in red color). These 4 unique candidates for nitration included secreted RXLR effector peptide protein (predicted), alcohol dehydrogenase (predicted), type I inositol-3,4-bisphosphate 4-phosphatase (predicted) and peroxiredoxin-like protein.

The comparative analyses of the obtained nitroproteome maps revealed that the *in planta* phase of *avr* MP 946 was accompanied by a lowered expression of 3 common spots (s1, s4, s5), whereas the presence of 2 spots (s2, s3) was no longer detected (Figure 3C). Interestingly, these reduced/absent nitrated proteins included proteins involved mainly in cellular redox homeostasis and signal transduction (Table 1). Additionally, the *in planta* phase of *avr* MP 946 revealed diminished expression of glyceraldehyde-3-phosphate dehydrogenase (s11).

Abundance profiles of nitrated proteins showed that 7 protein spots (s6–s10, s12, s13) were upregulated and 13 appeared *de novo* (s14–s26) during *in planta* hyphal growth of *avr* MP 946 (Figure 3C), however, only 4 of them (s10, s16, s17, s22) were common with *vr* MP 977 growing *in planta* (Figure 4C). These highly abundant spots included proteins involved in protein and carbohydrate metabolisms, signal transduction and pathogenesis (Table 1).

The qualitative nitroproteome analysis of the *vr* MP 977 *P. infestans* isolate resulted in the identification of a wider spectrum of nitrated proteins in comparison to the *avr* MP 946 (Figure 4). Nitroproteome maps revealed 32 spots during *in vitro* (Figure 4A) and 36 spots during *in planta* phases (Figure 4B). Twenty four protein spots were found to be common for both growth conditions. As many as 8 (s2, s3, s18, s21, s30, s33, s34, and s36) spots were specific to *in vitro* growth and 4 of them (s30, s33, s34, and s36 in red color) were indicated as exclusively nitrated in hyphae of *vr* MP 977 *P. infestans* growing *in vitro* (Figure 4A). In turn, during *in planta* growth we identified 12 (s37–s48) specific spots and all of them were marked as exclusively nitrated in hyphae of MP 977 *P. infestans* growing on potato (Figure 4B,C in gray color). The *vr* MP 977-specific candidates for nitration included two crinkler (CRN) family proteins, triosephosphate isomerase, prohibitin, serine/threonine protein kinase, syntaxin-like protein, choline/carnitine O-acyltransferase, 2-amino-3-ketobutyrate coenzyme A ligase (predicted), a mitochondrial ATP synthase subunit beta and three uncharacterized proteins.

Apart from the nitrated proteins common with *avr* MP 946 (s1–s5), analyses of the *vr* MP 977 isolate in the *in planta* phase showed a diminished expression of 16 other protein spots (s6–s9, s12–s15, s19, s20, s27–s29, s31, s32, s35), while the presence of 6 spots (s18, s21, s30, s33, s34, s36) was no longer detected (Figure 4C). These protein spots contain protein associated with carbohydrate/lipid metabolisms, oxidation-reduction processes and pathogenesis (Table 1).

Additionally, 5 spots (s10, s11, s16, s17, s22) were upregulated and 12 (s37–s48) appeared *de novo* in *vr* MP 977 when growing *in planta*. Interestingly, these 12 spots were found to be unique for *vr* MP 977 (Figure 4C) and the identified amino acid sequences (Supplementary Table S1) corresponded to the protein involved mainly in the processes of protein metabolism and signal transduction (Table 1).

### Identification and Metabolic Fate of the Differentially Expressed Nitrated Proteins of *P. infestans* During an Interaction With the Host

Post-translational modifications are some of the most important mechanisms activating, changing or suppressing protein functions in living organisms. Targeted nitration *via* ONOO^-^ could control protein structure and in consequence reversibly or irreversibly downregulate its activity (Yakovlev and Mikkelsen, 2010; Sengupta and Bhattacharjee, 2016). Alternatively, selective Tyr nitration regulates enzyme catalytic attributes or does not affect protein activity completely (Daiber et al., 2013). Based on this assumption, protein nitration may have biological consequences comparable with those of protein phosphorylation, i.e., may be involved in redox signaling (Kanski and Schoneich, 2005). Thus, the pool of nitrated proteins expressed in *P. infestans* could correspond to proteins in their inactive configuration reinforcing nitro-oxidative signaling.

Identification of immunoreactive spots, which expression *in planta* was downregulated in both *P. infestans* isolates, indicated NO-dependent events connected with primary metabolism, including nitration of proteins involved in the nucleobase-containing compound and carbohydrate metabolism, cytoskeleton organization, signal transduction and cell redox homeostasis. One of the identified nitrated proteins engaged in signal transduction was calmodulin (PITG_06514). It is well known that calmodulin is a calcium binding protein that serves as a molecular switch to regulate a network of the Ca^2+^ signaling pathway (Johnson, 2006). Importantly, recent studies indicate essential roles for cytoplasmic Ca^2+^ in the regulation of most aspects of oomycete biology, including pathogen virulence (Zheng and Mackrill, 2016). According to Zheng et al. (2018), the *P. infestans* RXLR effector SFI5 requires association with calmodulin for PTI suppressing activity. Thus, the substantially reduced pool of nitrated calmodulin (PITG_06514) *in planta* could favor the *P. infestans* offensive strategy. As it was also noted *in planta* in *vr* MP 977, the downregulated nitration of the transient receptor potential (TRP) channel superfamily (PITG_05737) and voltage-gated potassium channels (VGCC) (PITG_03719), which can act as Ca^2+^ channels, may have numerous effects on the regulation of oomycete Ca^2+^ signaling during host colonization. It is worth pointing that next to cell redox homeostasis, also heat shock proteins (Hsps) have been implicated in fungal pathogenicity. As it was indicated by Martinez-Rossi et al. (2017), Hsps display specific roles in fungi, such as dimorphic transition, antifungal resistance and virulence. Among the identified proteins in *vr* MP 977 showing a decreased nitration *in planta*, three were related to cell redox homeostasis. One of these proteins (PITG_14492) is a member of the NmrA-like family and could act as a redox sensor and regulator of transcription (Lamb et al., 2003). Thus, NmrA-like proteins might detect the degree of host defense response and contribute to the effective regulation of the pathogen’s offensive strategy. Importantly, the *avr P. infestans*-potato interaction was accompanied by elevated NmrA-like protein nitration.

It should be noted that energy metabolism is an important target for NO during *P. infestans in planta* growth. Diminished expression of nitrated proteins during *in planta* growth of *vr* MP 977 involved many metabolic regulators, including enzymes that may mobilize reserves and maintain energy homeostasis. These proteins included triosephosphate isomerase, aspartate aminotransferase, carbonic anhydrase, pyruvate kinase, inositol-3-phosphate synthase and 3-ketoacyl-CoA thiolase. Thus, the decreased expression of nitrated proteins involved in carbohydrate and lipid pathways during *in planta* growth might accelerate energy and metabolic intermediates in *P. infestans* infection structures. Moreover, in order to export cell wall components and defend against host-derived toxic compounds plants need to mobilize stored energy reserves (Resjö et al., 2017). Importantly, five other nitrated proteins engaged in energy metabolism were either found in greater amounts or appeared *de novo*.

Four of the NO-regulated proteins identified in both *P. infestans* isolates during the *in planta* phase were involved in nucleic acid metabolisms. Two of these proteins, i.e., nitrated ATP-dependent RNA helicase SUPV3L1 and the structural maintenance of chromosome protein were oppositely expressed in the analyzed isolates and upregulation of these proteins was observed only in *avr* MP 946. This could reflect the importance of this protein group as the main target for nitration during interactions with the plant. The relevance of the proper metabolism regulation of nucleobase-containing compounds for pathogen survival within the host was postulated earlier, since defects in nucleic acid metabolism may lead to an inappropriate activation of nucleic acid sensors (Lee-Kirsch, 2010; Sharma et al., 2015).

Among the identified nitrated proteins related to signal transduction, four showed an increased expression or appeared *de novo* in both isolates, however, only guanine nucleotide-binding protein subunit beta-2-like protein (PITG_09556) revealed common upregulation. This PTM may result in synergistic or antagonistic relations between signaling hubs and modulate signal transduction attenuating pathogen offensive.

Several proteins related to pathogenicity *sensu stricto* were also found to be nitrated in *P. infestans*. The genome of *P. infestans* is one of the largest among oomycetes (240 Mb), containing an extremely large pool of predicted genes coding for disease-promoting effector proteins, leading to an exceptionally high potential to adapt to new control strategies of the potato crop. In *P. infestans* these predicted host-translocated effectors encompass the RXLR (containing the conserved motif RXLR for arginine, any amino acid, leucine, and arginine) and CRN (crinkler or crinkling- and necrosis-inducing protein) effectors. These are large and complex protein families, with around 560 RXLRs and 200 CRNs members encoded in the genome (Meijer et al., 2014), allowing oomycetes to manipulate plant immunity and promote infection. Some of the RXLR and CRN effectors have been experimentally verified, and their function as virulence and avirulence factors have been elucidated (Haas et al., 2009). Importantly, we detected a relatively large quantity of potentially nitrated cytosolic RXLR (5 identified) and CRN (2 identified) effectors, which were differentially expressed during *in planta* growth. In the case of the *avr* MP 946 isolate, nitration of RXLRs (PITG_23137, PITG_06059) was upregulated, whereas in *vr* MP 977 all identified RXLRs (PITG_01904, PITG_04081, PITG_22813) exhibited downregulated expression. Moreover, the *vr P. infestans*–potato interaction resulted in nitration of two CRN effectors (PITG_23273, PITG_20172). Since *P. infestans* has the capacity to rapidly change its repertoire of effectors and thereby escape recognition by resistant potato varieties, the PTM of effector proteins seems to be a promising signpost for further analysis. NADH dehydrogenase [ubiquinone] flavoprotein 1 (PITG_06815) was one of the pathogenicity-related proteins that were more abundant in both isolates growing *in planta*. Moreover, another putative pathogenicity related protein included actin like protein (PITG_15078), playing an important role during appressorium formation in this filamentous organism (Kots et al., 2017).

Intriguingly, three of the proteins that we found to be nitrated, were also identified as potentially phosphorylated on Tyr residues (Resjö et al., 2014). These include actin-like proteins (PITG_15078; s12), heat shock 70 kDa protein (PITG_11244; s32) and serine/threonine protein kinase (PITG_14221; s44). Several reports point to a dynamic interplay between protein Tyr nitration and phosphorylation, which is dependent on the concentration of nitrating agents in the biological milieu. Competition between phosphorylation and nitration occurs within the same sensitive Tyr residue. While Tyr phosphorylation is induced at lower concentrations of peroxynitrite, at relatively higher amounts the process is inactivated and it is also irreversible (Sengupta and Bhattacharjee, 2016). Although the competition correlation between phosphorylation and nitration is as yet poorly understood, it might partially explain the dynamic change in nitroproteome during the switch between *in vitro* and *in planta* pathogen growth.

## Conclusion

To our knowledge, presented findings contribute further insight into *P. infestans* molecular biology and for the first time indicate protein Tyr nitration as a common phenomenon within pathogen structures, engaged in the regulation of metabolic activities under *in vitro* and *in planta* conditions. Thus, the nitroproteome should be considered as a flexible element of the oomycete adaptation strategy to different micro-environments.

Definitely, due to the increased awareness of fungicide effects and the phasing out of various effective crop protection compounds, the identification of NO-mediated functional modifications may contribute to improvements in modern potato protection strategies against late blight disease.

## Author Contributions

MA-J and JF-W planned and designed the research. KI performed the experiments. KI, JGa, and JGz collected and analyzed the data. TJ performed the statistical analysis. MA-J wrote the manuscript.

## Conflict of Interest Statement

The authors declare that the research was conducted in the absence of any commercial or financial relationships that could be construed as a potential conflict of interest.

## Supplementary Material

FIGURE S1 The index of disease development on potato leaves at 9 dpi with *avr* MP 946 and *vr* MP 977 *P. infestans* based on a I–IV point scale, which represents the percentage of leaf area covered by late blight symptoms. Values are means of average disease indexes from 10 leaves from three independent experiments.

FIGURE S2 Tyrosine nitration pattern of *avr* MP 946 **(A)** and *vr* MP 977 **(B)** *P. infestans* growing *in vitro* and *in planta* conditions, using 10 mM sodium dithionite as control of anti-nitrotyrosine antibody specificity. Treatment PVDF membranes with sodium dithionite (prior to incubation with the polyclonal antibody against nitrotyrosine diluted at 1:1000) converts nitrotyrosine in aminotyrosine, allowing the detection of false positive spots. Molecular-mass standards (kDa) are indicated on the left.

TABLE S1 Amino acid sequence of the identified homologous proteins (matched peptides derived from *P. infestans* shown in bold red).
