# Supplementary figures and images for: Switchable Nitroproteome States of Phytophthora infestans Biology and Pathobiology
Source: Front Microbiol. 2019 Jul 16;10:1516. doi: 10.3389/fmicb.2019.01516 (PMC6647872; doi:10.3389/fmicb.2019.01516)

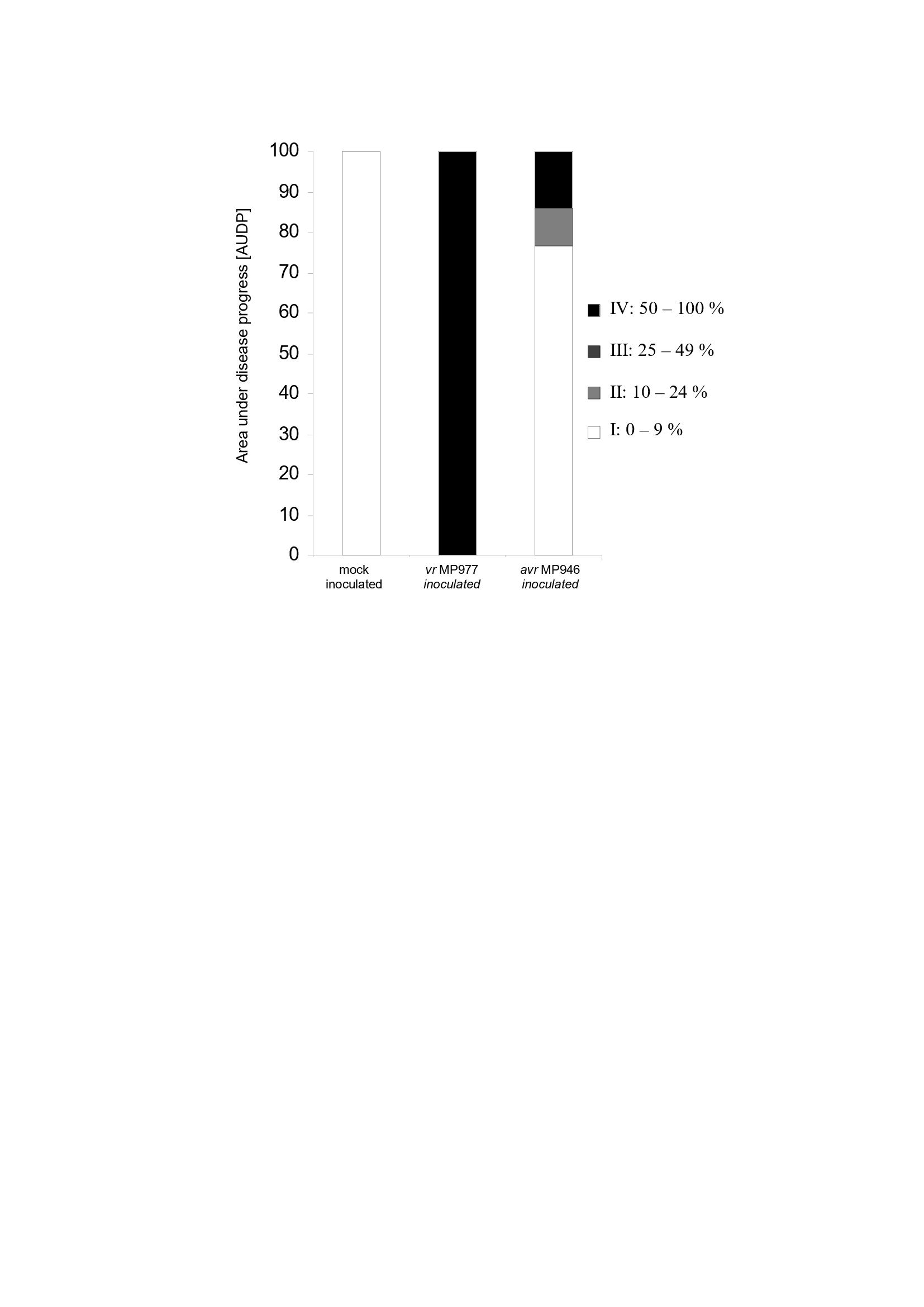

Supplement: FIGURE S1 — The index of disease development on potato leaves at 9 dpi with avr MP 946 and vr MP 977 P. infestans based on a I–IV point scale, which represents the percentage of leaf area covered by late blight symptoms. Values are means of average disease indexes from 10 leaves from three independent experiments. [file Image_1.JPEG]

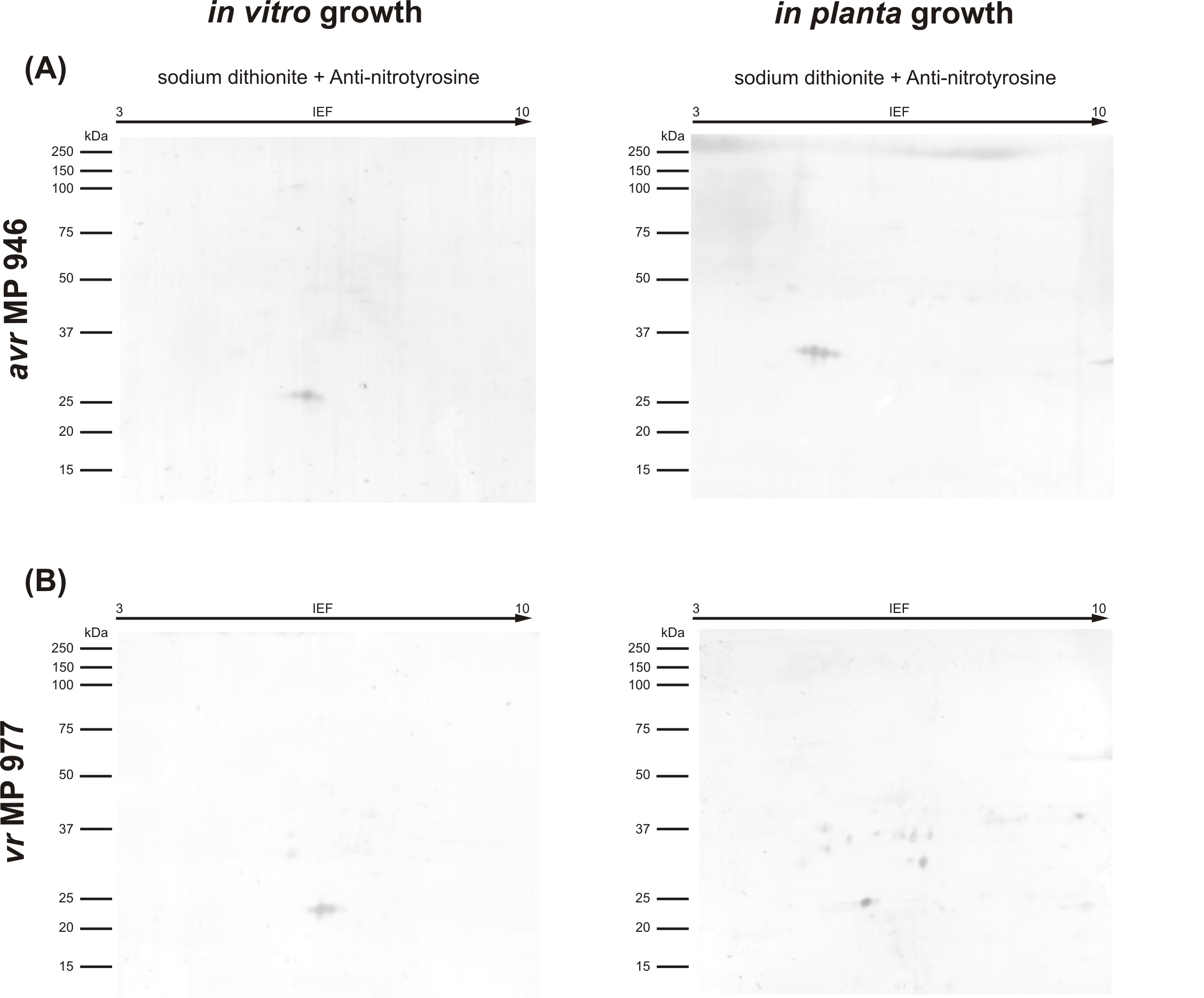

Supplement: FIGURE S2 — Tyrosine nitration pattern of avr MP 946 (A) and vr MP 977 (B) P. infestans growing in vitro and in planta conditions, using 10 mM sodium dithionite as control of anti-nitrotyrosine antibody specificity. Treatment PVDF membranes with sodium dithionite (prior to incubation with the polyclonal antibody against nitrotyrosine diluted at 1:1000) converts nitrotyrosine in aminotyrosine, allowing the detection of false positive spots. Molecular-mass standards (kDa) are indicated on the left. [file Image_2.TIF]
