# Supplementary material for: Switchable Nitroproteome States of Phytophthora infestans Biology and Pathobiology
Source: Front Microbiol. 2019 Jul 16;10:1516. doi: 10.3389/fmicb.2019.01516 (PMC6647872; doi:10.3389/fmicb.2019.01516)
Supplement: TABLE S1 — Amino acid sequence of the identified homologous proteins (matched peptides derived from P. infestans shown in bold red). [file Table_1.DOCX]

Table S2 Amino acid sequence of the identified homologous proteins (matched peptides derived from *Phytophthora infestans* shown in **Bold Red**)

| No. | NCBI  accession no. | Matched peptides (**BOLD RED**) |
| --- | --- | --- |
| **S1** | **PITG_12377** | \| 1 \| MWTYRGDPYV \| PTFVVEIDEL \| SGPGSQLSAL \| DRKMRNEYFQ \| HGVQLGWLID \| \| --- \| --- \| --- \| --- \| --- \| --- \| \| 51 \| PRPD**LQRMYE** \| **YYLDDN**GDVQ \| CSDNSVWRDL \| DGGDVLPGFK \| MRAPVLEMVL \| \| 101 \| NQDSGSSSED \| EVDLLCPSPR \| CNKRF**RSYGA** \| **CAAHVEW**HRK \| ERSISKYLAK \| \| 151 \| RENS \|  \|  \|  \|  \| |
| **S2** | **PITG_03552** | \| 1 \| MSGRGKGGKG \| LGKGGAKRHR \| KVLRDNIQGI \| TKPAIRRLAR \| RGGVKRISGL \| \| --- \| --- \| --- \| --- \| --- \| --- \| \| 51 \| IYEE**TRGVLK** \| **VFLENVIR**DS \| VTYTEHARRK \| TVTAMDVVYA \| LKRQGRTLYG \| \| 101 \| FGG \|  \|  \|  \|  \| |
| **S3** | PITG_07996 | \| 1 \| MREVISIHLG \| QGGIQVGNAC \| WELYCLEHGI \| QPDGQMPSDK \| TIGGGDDAFN \| \| --- \| --- \| --- \| --- \| --- \| --- \| \| 51 \| TFFSETGAGK \| HVPRAVFVDL \| EPSVCDEVRT \| GTYRQLYHPE \| QIISGKEDAA \| \| 101 \| NNYARGHYTI \| GKEIVDLVLD \| RIRKLADNCT \| GLQGFLVFNA \| VGGGTGSGLG \| \| 151 \| ALLLERLSVD \| YGRKSKLGFT \| IYPSPQVSTA \| VVEPYNSVLS \| THSLLEHTDV \| \| 201 \| AVMLDNEAIY \| DICRRSLDIE \| RPTYTNLNR**L** \| **IAQVISSLTA** \| **SLR**FDGALNV \| \| 251 \| DVTEFQTNLV \| PYPRIHFMLS \| SYAPVISAEK \| AYHEQLSVAE \| ITNSAFEPAS \| \| 301 \| MMAKCDPRHG \| KYMAACLMYR \| GDVVPKDVNA \| AVATIKTKRT \| IQFVDWCPTG \| \| 351 \| FKCGINYQPP \| TVVPGGDLAR \| VQRAVCMISN \| TSAIAEVFSR \| IDHKFDLMYA \| \| 401 \| KRAFVHWYVG \| EGMEEGEFSE \| AREDLAALEK \| DYEEVSAETA \| EGEGEEEELG \| |
| **S4** | **PITG_06415** | \| 1 \| MSAAEAETFA \| FSADINQLLS \| LIINTFYSNK \| DIFL**RELISN** \| **ASDALD**KIRY \| \| --- \| --- \| --- \| --- \| --- \| --- \| \| 51 \| SSLTDASVLD \| TDKNLEIKVT \| PDKANGTLTI \| QDSGIGMTKA \| DLINNLGTIA \| \| 101 \| KSGTKAFMEA \| LAAGADISMI \| GQFGVGFYSA \| YLVADKVVVH \| SKHNDDEQYV \| \| 151 \| WESAAGGSFT \| VTPDTSEPIQ \| RGTRIVLKLK \| ED**MLEYLEER** \| **KLK**DLVKKHS \| \| 201 \| EFIGFPIKLY \| VEKTEEKEVT \| DDEEEEDEKE \| GEDDKPKVEE \| VEEEEGEKKK \| \| 251 \| KTKKIKEVTH \| EWDHLNSQKP \| IWMRKPEDVT \| HEEYASFYKS \| LTNDWEEHAG \| \| 301 \| VKHFSVEGQL \| EFKACLFTPK \| RAPFDMFEGG \| AKKKVNNIKL \| YVRRVFIMDN \| \| 351 \| CEELMPEYLS \| FVKGVVDSED \| LPLNISRETL \| QQNKILRVIK \| KNLVKKCLEM \| \| 401 \| FAELAEDNEK \| YNKFYESFSK \| NLKLGI**HEDS** \| **TNRTKIAKL**L \| RYHSTKSGEE \| \| 451 \| VTSLDDYISR \| MPESQPGIYY \| VTGESKKSVE \| NSPFIEKLKK \| KGYEVLFMVE \| \| 501 \| AIDEYAVQQL \| KEYEGKKLIC \| ATKEGLKMEE \| TEDEKKSFEE \| AKAATEGLCK \| \| 551 \| LMKEVLDDKV \| EKVEISNRIV \| ESPCVLVTGE \| YGWSANMERI \| MKAQALRDSS \| \| 601 \| TSAYMSSKKT \| MEINPLHPII \| KSLREKAEAD \| KSDKTVKDLI \| WLLYDTSLLT \| \| 651 \| SGFSLDEPTT \| FANRIHRLIK \| LGLSIDDDDD \| AADESMEDLP \| PLEGEDEEES \| \| 701 \| TMEEVD \|  \|  \|  \|  \| |
|  | PITG_19017 | \| 1 \| MDRDSLVFLA \| K**LAEQAERYD** \| **EMVDH**MKAVA \| NNHNVELTVE \| ERNLLSVAYK \| \| --- \| --- \| --- \| --- \| --- \| --- \| \| 51 \| NVIGSRRASW \| RVISSIENKG \| DSERS**EHIKA** \| **YRQKIEGELV** \| **DICNDILTI**I \| \| 101 \| ENNLIPNSSS \| EEGKVFYYKM \| KGDYHRYLAE \| FQVDDERKES \| SDKALESYKQ \| \| 151 \| ASTIAMAELP \| PTHPIRLGLA \| LNFSVFYYEI \| LNSPDRACNL \| AKQAFDDAIA \| \| 201 \| ELDTLSEESY \| KDSTLIMQLL \| RDNLTLWTSD \| QEPDNADANQ \| GDMNVQDVE \| |
|  | PITG_06514 | \| 1 \| MADQLTEEQI \| AEFKEAFSLF \| DKDGDGTITT \| KELGTVMR**SL** \| **GQNPTEAELQ** \| \| --- \| --- \| --- \| --- \| --- \| --- \| \| 51 \| DMINEVDADG \| NGTIDFPEFL \| TMMARKMKDT \| DSEEEILEAF \| KVFDKDGNGF \| \| 101 \| ISAAELRHIM \| TNLGEKLTDE \| EVDEMIREAD \| IDGDGQINYE \| EFVKMMMSK \| |
| **S5** | PITG_02786 | \| 1 \| MGLLDIVQPG \| VLNGEDVVKV \| YKYAQEHNFA \| IPAVNVTSSS \| TVNAALQAAR \| \| --- \| --- \| --- \| --- \| --- \| --- \| \| 51 \| DIK**SPIIIQT** \| **SNGGAAFYAG** \| **K**GIDNKNQNG \| SILGAIAAAY \| HVRAMAKHYG \| \| 101 \| VPVILHSDHC \| AKKLLPWYDG \| MLEADEKYFA \| EHGVPLWSSH \| MLDLSEEPME \| \| 151 \| ENVAISKKYF \| ERMAKMNLIL \| EVELGITGGE \| EDGVDNSDVD \| NASLYSQPED \| \| 201 \| ILLAYNELGS \| VSPYFTIAAA \| FGNVHGVYKP \| GNVK**LHPEIL** \| **GDFQK**YVAKE \| \| 251 \| KSLSTEKPVF \| FVFHGGSGST \| PQEIQTAVGN \| GVVKMNIDTD \| TQWAYWNGLR \| \| 301 \| **KFYEEK**K**DYL** \| **QGQIGNPDGP** \| **DAPNK**KYYDP \| RVWVR**KSEET** \| **MIARLHEAFR** \| \| 351 \| DLNCVNVL \|  \|  \|  \|  \| |
|  | PITG_03551 | \| 1 \| MARTKQTARK \| STGGKAPRKQ \| LATKAAR**KSA** \| \| **PATGGVK**KPH \| RYRP**GTVALR** \| \| --- \| --- \| --- \| --- \| --- \| --- \| --- \| \| 51 \| **EIR**RYQKSTE \| LLIRKLPFQR \| LVREIAQDFK \| \| TDLRFQGSAV \| LALQEAAEAY \| \| 101 \| LVGLFEDTNL \|  \| \| |
|  | PITG_15492 | \| 1 \| MAPKIRHAAP \| AFTADAVVDG \| EFKTVSLSDY \| KGKYVVLFFY \| PMDFTFVCPT \| \| --- \| --- \| --- \| --- \| --- \| --- \| \| 51 \| EIIAFSEKAA \| EFRKLGCEVL \| GCSVDSKFSH \| LAWINTPRKQ \| GGLGELDIPL \| \| 101 \| LADFNKEISQ \| AYDVLIDVGE \| ETGATFRGLF \| IIDGEGKLR**Q** \| **STINDCPVGR** \| \| 151 \| NVDEILRLVE \| AFQFTDEHGE \| VCPAGWKKGK \| KTIKPTVDAS \| KEYFEAAN \| |
| **S6** | **PITG_11795** | \| 1 \| MSKYSKTYLA \| LAPVADPTAR \| EHLLHAAAPA \| IDAGTPINDD \| FLLSARIERQ \| \| --- \| --- \| --- \| --- \| --- \| --- \| \| 51 \| LREIEAQRGM \| VTRHEVLAAT \| IREHAILMEH \| AEVEYPKAVA \| PTVMPSAQLI \| \| 101 \| PNSIYKPSYA \| QVTHCIPSQR \| HFSSSAVEAE \| LQSVKWPSDL \| AKRVHRRMRS \| \| 151 \| FPRNYEAKEL \| VVEAGMNQAE \| WAIAAASFRK \| SFLSEPSKYF \| KNQQE**LLAFG** \| \| 201 \| **RDLDDVK**RHN \| SFIFYPYFVD \| YAKANSYLPT \| DQDNDNVLSL \| QQLTDLRLPH \| \| 251 \| EMYPFATAMK \| RKIIYHEGPT \| NSGKTHQALE \| RLKQAGEDGG \| IYCGPLRLLA \| \| 301 \| LEIFERLNAD \| GLYTSLVTGQ \| EKKLVPYSTH \| VSCTVEMANI \| NRPWDVAVVD \| \| 351 \| EIQLIGDPQR \| GWAWTRALFG \| LQANEIHVCG \| SGEAVHLVKK \| FAETTGDDFE \| \| 401 \| LRSYERRSPL \| EIAPTHLASY \| SHIRSGDCVV \| AFSRRDIFQI \| KRDIEVKTGQ \| \| 451 \| KCCIIYGQLP \| PETRSQQARL \| FNDRNNDFNI \| LVASDAIGMG \| LNLNIRRVVF \| \| 501 \| ATVK**KYSGSS** \| **GGMID**IPPSL \| AKQIAGRAGR \| YGSDFASGEA \| TCVLEEDLEY \| \| 551 \| LKESYDEVPT \| PLTSAGLFPS \| SEQMEEFARQ \| LPGITDLADL \| VDKYVMLARL \| \| 601 \| DGDYFMCNHQ \| DMKDAATLLR \| ETELTLSDRF \| TFCMSPVGLR \| NPLARRVFLE \| \| 651 \| YARAHSLGQS \| VRLDIYLPKY \| APRTAEALGD \| VEIKAKIIDL \| YLWLSFRFED \| \| 701 \| TFVEKDLALE \| LKTRVLELVE \| QGLVNTTYHR \| EDK**KTRWSSG** \| **AANGRRSASR** \| \| 751 \| **GQF**DGDRFRQ \| QTKDGDSRPN \| WRRDKTTRQP \| AQASRRTNTQ \| REDGAEGLKE \| \| 801 \| TEDSNMSWVA \| RMFGTSK \|  \|  \|  \| |
| **S7** | **PITG_00319** | \| 1 \| MAMSFIWSLV \| HACREGMLCY \| SWLMSKVDEQ \| FLNEGACALA \| KRM**VDAWPFE** \| \| --- \| --- \| --- \| --- \| --- \| --- \| \| 51 \| **RLPGF**GEGFD \| LDWTHLYCAR \| SECWYHDSLI \| NALMMTLAEN \| FKNNTTLFLP \| \| 101 \| PLYTPAPSKC \| KRIPPRTLSL \| VAAADKDLVF \| MPLNINGKHW \| VCLVLDRPRT \| \| 151 \| TIYCYDSFDK \| RSNQIMLAEL \| AEEIGRKALS \| DSDNCGLFII \| LHFW**RRFVKE** \| \| 201 \| **MGSDYT**TVGL \| LRRRWDVLRT \| VVDFSDASKG \| EQD \|  \| |
| **S8** | PITG_05757 | \| 1 \| MGRIARLELE \| NFKSYGGYHV \| VGPFHRFTAV \| IGPNGSGKSN \| LMDAISFVLG \| \| --- \| --- \| --- \| --- \| --- \| --- \| \| 51 \| VHSRQLRSNQ \| LRDLVHKAPT \| DTATTGRSAF \| VTLVYELSAD \| ETPPSKSLAA \| \| 101 \| QNQQKEVKFT \| RLISEKGAGS \| YRIDGQDVSS \| EGYQNQLKEI \| GILVKSRNFL \| \| 151 \| VFQGEVESIA \| SKSPTELTKL \| FEQISMSDEL \| KNEYERLMEE \| KDAAEESTIF \| \| 201 \| AYKRKKGLVA \| EKRLVREQKE \| EAEQFRHKQD \| AVNDLRVEHY \| LWQLFQVEDD \| \| 251 \| MTQREETVRQ \| YQGARRTCSQ \| KEEDVAQTYR \| EKKKELNASL \| REVKTNRKRI \| \| 301 \| QDLQSEMEDI \| QPQVIRLREQ \| TQYSQRKIVE \| SETTEKQMKE \| RQEGKAKEIE \| \| 351 \| GLKTDLQELE \| KVKAELEAKQ \| AKEASQRGEE \| GSLVLEGSRL \| DEYHRIKEAV \| \| 401 \| QVKTNLLRNE \| LESILRQQNA \| DKNKVETLSQ \| ERQENLKMIE \| MLSDDLKQAD \| \| 451 \| ERVVSMQCVI \| SDTERDIADA \| EKSLQTADDE \| KRGQAEEKEK \| LTKQLERVNN \| \| 501 \| KLRDLKDDKR \| QSQAEARRAD \| TLETLKRLYP \| GVRGRLVDLC \| KPTQRKYNMA \| \| 551 \| VTVATGKHMD \| AIVVTDYRTG \| QECIQYLRDS \| RAGSAQFIPL \| DKIRVKPINE \| \| 601 \| RFRGLGNNIK \| MVVDVVQCDP \| ENEPALHYAV \| GDTVVCETIE \| VARDLCFRQN \| \| 651 \| EKLKAVTLNG \| MVVSKNGSMT \| GGKTQNDLRR \| AGRWDEKEVE \| ALQQEKDKLI \| \| 701 \| DAIRAIERHG \| ASYAKLQTQR \| TQIEGLKSRL \| THAKADLVIT \| ENKRPKIQLR \| \| 751 \| IDEAKKRVSE \| VIEPELGKFA \| AAVESRRAKI \| DALQDQIHGV \| EDEMFADFSE \| \| 801 \| AIGVDSIRVY \| EERVLKRHHK \| AMEMRRKITE \| HEAKLRAQIE \| YLESQDFNQP \| \| 851 \| MLAARERATR \| EAQHLKTLVE \| EEAALMKTFA \| VLRKERKEHE \| ALRQTLSTKV \| \| 901 \| EELEKALREI \| GSKKAKYEQR \| KGKIQRRISS \| EETVLERLKD \| HKTELFK**RAA** \| \| 951 \| **LDQIK**LPTVA \| RSGSEDIEME \| DASASSSLEN \| TELLLGADAA \| NRQVDFSSLS \| \| 1001 \| DAHVVVDDKE \| FDEINADYEK \| RIGLLLTELE \| QIQPNMRALD \| KFDVIQSRIG \| \| 1051 \| KEEEELDRIK \| QQALDTASKF \| EKVKQTRRDR \| FMEAFTHISG \| VIDSTYKQFT \| \| 1101 \| KSSKHPLGGT \| AYLNLENTEE \| PYLSGMKFNA \| MPPMKRFREM \| DELSGGEKTV \| \| 1151 \| AALALLFAIH \| NYRPSPFFVL \| DEVDAALDNV \| NVNKVSTYIA \| NCGFQCVVIS \| \| 1201 \| LKDSFYEKAD \| ALVGVCRDIT \| LQQSKSMTLD \| LTKFD \|  \| |
| **S9** | PITG_12490 | \| 1 \| MRSP**VTLLLL** \| **TLSFIFFNGE** \| EEATTATTEV \| SPESPWGRIY \| LRNGLAPIQY \| \| --- \| --- \| --- \| --- \| --- \| --- \| \| 51 \| LRDDFGGPMT \| SREVSLYFPK \| RRKNRFGCEL \| LPESESMEVE \| AANRSVVLVV \| \| 101 \| DRGECTFEH**K** \| **ALLADQMGAA** \| **ALLVVS**PTDD \| VSAPVAALKN \| DEEISIASVM \| \| 151 \| IRRTGGDMLR \| IAAEQMTIYG \| RFIPMTCERK \| P**YTCKPRYAD** \| **EEDYI**ESAIA \| \| 201 \| RSGVVLSVDK \| KGDDGTQVGT \| FLAATYGSVL \| PTKMPFLLAA \| PLDGIQACTD \| \| 251 \| ATKDSATHSE \| FEGKVVLIPA \| GLAGKCSEFE \| KVSNAQRRGA \| NVVVLMQQDN \| \| 301 \| ATITTQPGVP \| VSWHAYNITI \| PVLAVSSTTG \| ANLAGLKDAH \| GDAARLRFAV \| \| 351 \| SNGIADAWEL \| LKQYSTRSSW \| PKRVKRCSKT \| LAQLLYQLRG \| FGGDIEVEEA \| \| 401 \| LKSMFLNVVS \| GSLQDWEKIA \| HPDQDADQDH \| ETQKRSSSEK \| IVQTVEKSET \| \| 451 \| RDEF \|  \|  \|  \|  \| |
| **S10** | PITG_06815 | \| 1 \| MKSVARVAQK \| TLSQARGFRT \| SAAVANAAEE \| PAMARVYGGL \| KDEDRIFTNV \| \| --- \| --- \| --- \| --- \| --- \| --- \| \| 51 \| YGEDTWRVDG \| AVRRGDWHRT \| KDLLCMGPDW \| IVQEIKDSGL \| RGRGGAGFPS \| \| 101 \| GLKWSFMPKQ \| TDGRPSFLVV \| NADESEPGTC \| KDREIMRKDP \| HKLVEGCLIA \| \| 151 \| GFAMRARAAY \| VYIRGEFFNE \| ALILQEAIHE \| AYQRGFLGRN \| CGSGYDFDV \| \| 201 \| YLHRGAGAYI \| CGEETGLLES \| LEGRQGKPRL \| KPPFPANTGL \| YGCPTTVTNV \| \| 251 \| ETVAVSPTIL \| RRGGSWFASL \| GRK**NNHGTKL** \| **FCISGH**VNNP \| CTVEEEMSIP \| \| 301 \| LRDLIERHCG \| GVRGGWDNLQ \| ACIPGGSSVP \| VLDEELCQDI \| MMDYDDLKQR \| \| 351 \| GGSGLGTAAV \| TIFDKSVDMV \| GAIRRYSHFY \| THESCGQCTP \| CREGTGWMEK \| \| 401 \| VLTRMETGDA \| ELEEIPMLEE \| ISRQIEGHTI \| CALGDAAAWP \| VQGLIRK**FKH** \| \| 451 \| **KLVERIED**PS \| SFKPEDYFQK \| AWPGAPLKNQ \| EWVDKFADGS \| AYKSQASA \| |
| **S11** | PITG_01938 | \| 1 \| MVAINDPFMD \| LEYMAYLFKY \| DSTHGK**FDGS** \| **VATKDGNLVI** \| **NGEVIHVFTA** \| \| --- \| --- \| --- \| --- \| --- \| --- \| \| 51 \| **RNPAEIPWGK** \| **AGATYVCEST** \| **GVFTTTEK**AQ \| SHIEGGCKKV \| VISAPPK**DST** \| \| 101 \| **PMYVMGVNHK** \| **EYDGSAPVVS** \| **NASCTTNCLA** \| **PLAKVINDNF** \| **GIVEGLMTTV** \| \| 151 \| **HATTATQLPV** \| **DGPAK**GGKDW \| RGGR**GCGQNI** \| **IPSSTGAAK**A \| VGKVLPVLNG \| \| 201 \| K**LTGMAFRVP** \| **TPDVSVVDLT** \| **CR**LEKAASMD \| AIKAAVKAAS \| EGELAGILGY \| \| 251 \| TEDQVVSNDF \| LHDKHSSTFD \| ADACIALNDH \| FVKLVAWYDN \| EWGYSNRLVD \| \| 301 \| LVLHMATVDI \|  \|  \|  \|  \| |
| **S12** | PITG_06853 | \| 1 \| MQRGLLRLSR \| AALNNHQHVP \| TAIRTISSTP \| PNSGPGRRRP \| SRTQVIDDIG \| \| --- \| --- \| --- \| --- \| --- \| --- \| \| 51 \| ALVHEAKRQG \| PRFQPSMDYL \| R**RASAVLQLC** \| KGREQCTAAI \| PLCHIVEKAQ \| \| 151 \| NTHSKAEMEC \| VRIYQKAGRN \| QEVVALAEKL \| MSQDVFLLNP \| ALASAIQACA \| \| 201 \| AVGDM**DKGFH** \| **FFDVAVKRG**S \| IPNLSVYSAL \| LAVAGATGDP \| KRVQTVLTQM \| \| 251 \| QDAGVEMNDI \| TFHNLMSAYA \| RGGHVTEALA \| LF**DKMQKQGI** \| **PADEHTY**AIL \| \| 301 \| **MDAHAESGDF** \| **EGANALMEEL** \| KKASIGPNLV \| HYNVLLKACG \| KVRNLTSAFQ \| \| 351 \| LYEEMKERKI \| QPDLVTFITM \| MHAVYHGELG \| AVDQKKVKAA \| LVGMGVMGAA \| \| 401 \| FIPFINYEEY \| MMTTLFCGSL \| VGSMGLAAYM \| NPDGVI**RALY** \| **PNTDEPRD**DT \| \| 451 \| IIEAFFRRLR \| EEDHSGRSMY \| LWREMLKFNV \| PADPRVYDVL \| VRTCVRKRHP \| \| 501 \| ELAYEAVFEE \| KLPLTDKEGS \| FVLNLPTTLS \| LHSLLAQKR \| FN**MADTLYDA** \| \| 551 \| **ARGHGVFDKV** \| **FSEKGD**VYVY \| DMRSFLNEQV \| RSYAIIKLLD \| ELRTKVDSSK \| \| 601 \| GQFVAPNVQF \| LVQHGYELLD \| RLDADNTSLR \| TLFSMDDMAR \| IGASGDNSTS \| \| 651 \| ANYMYYFRLA \| IPTERLQKYF \| ETTPANAGKE \| RRL \|  \| |
|  | PITG_15078 | \| 1 \| MDDDIQAVVI \| DNGSGMCK**AG** \| **FAGDDAPR**AV \| FPSIVGMPKH \| LGIMVGMNQK \| \| --- \| --- \| --- \| --- \| --- \| --- \| \| 51 \| DAYIGDEAQA \| KRGVLTLRYP \| IEHGIVTNWD \| DMEKIWSHTF \| YNELR**VAPEE** \| \| 101 \| **HPVLLTEAPL** \| **NPK**ANRERMT \| QIMFETFNVP \| AMYVNIQAVL \| SLYASGRTTG \| \| 151 \| CVLDSGDGVS \| HTVPIYEGYA \| LPHAIVRLDL \| AGRDLTDYMM \| KILTER**GYSF** \| \| 201 \| **TTTAER**EIVR \| DIKEKLTYVA \| MDFDEEMEKS \| TRSSALDKTY \| ELPDGNVIVI \| \| 251 \| GNERFRTPEV \| LFNPSMIGRE \| CSGVHECAFQ \| TVMKCDVDIR \| RDLYSNVVLS \| \| 301 \| GGSTMFPGIG \| ERMTKEVIKL \| APTAMKVKII \| TPPERKYSVW \| IGGSILASLA \| \| 351 \| TFQHMWISKT \| EYDESGPSIV \| HRKCF \|  \|  \| |
| **S13** | PITG_02284 | \| 1 \| MPPHSATPSS \| PVATAGVRRD \| LLLYVNGERI \| EISERDVHPE \| QTLLQFLRHD \| \| --- \| --- \| --- \| --- \| --- \| --- \| \| 51 \| LGLAGTKLGC \| GEGGCGACTV \| MVSKFDVATG \| RVRHVSVNSC \| LAPFCAMDTC \| \| 101 \| AVTTVEGVGT \| ITGATGEATG \| LHEVQKVLAE \| SHASQCGYCT \| PGFVMALYSM \| \| 151 \| VKQRESGVEL \| TMEDIEHGMD \| GNLCRCTGYR \| PILDAAKSFG \| DDAGEAHCKG \| \| 201 \| TCPGCPNAKN \| GDADVDIEDL \| HGDNHQEVTS \| CSSRKIRELA \| KHCQLREKHD \| \| 251 \| VDTVTGASKN \| TKALAVSSFP \| NELMEKAMAP \| QTLQIDGKYI \| QWFAPVTITH \| \| 301 \| LLQLKKQHPD \| AKISVGNTEM \| GIETKFKGFK \| YAHLINVSRI \| PELVATKDVT \| \| 351 \| QTDPINQTVF \| SGAEPFEGVK \| LGAAVTLTDV \| KQQLSELIKT \| MPVYQTRAFE \| \| 401 \| SIVKMLKWFA \| STHIRNVACI \| AGNLVTASPI \| SDMNPLLAAM \| NAYIELQSTR \| \| 451 \| GTQYTRVRDF \| FLSYRKVGME \| PDEIITAVYV \| PYTKKWEYML \| PFKQARRRED \| \| 501 \| DISIVTAGIR \| VRLECSGDND \| AWIIQDASAV \| YGGMAPITKS \| AAETEQFLIG \| \| 551 \| KTFNASTFGE \| ACDVLHSSDF \| ELPDGVPGGM \| AKYRESLCSS \| FLYKFYVASS \| \| 601 \| ERLQLDLQAI \| KATGSLLSDA \| PVVDSTMQSA \| GTSFLHQVRP \| VSHGTQRFGR \| \| 651 \| ETGGLQDSKH \| QPIGDAKTKR \| GPVGDPLMHK \| SAYLQVSGEA \| LYTDDIPNTP \| \| 701 \| GTLHGALVLS \| TCAHGLIKSI \| DASEALAMEG \| VHRFFDASVF \| ETEKLGSNKI \| \| 751 \| GPVLKDEECF \| ASKEVLCVGQ \| PVGIIIADTH \| ELAMAASDQV \| QVVYEEL**PSV** \| \| 801 \| **TTIEEAIREK** \| **SFILPAHTIN** \| **SGNVE**TGLAE \| SDIVLEGEVH \| MGGQEQFYFE \| \| 851 \| TNVSLCTPQE \| GGMKVISSTQ \| AATKAQVLVA \| RVLGINSNRI \| TSTTKRIGGG \| \| 901 \| FGGKETRTVF \| VTCAAAVASH \| VMKRPVKCLL \| ERHVDMLTTG \| GRHPFYAKYK \| \| 951 \| VGIKQDGTIL \| ALDVDIYNNA \| GYSMDLSLAV \| MDRALFHCEN \| AYKIPNLRCH \| \| 1001 \| GTVCRTNLAT \| NTAFRGFGGP \| QGLFIAETYI \| DHIARTLKLS \| PEDVRTRNMY \| \| 1051 \| VEGQTTHFGQ \| PLEDFNLRTL \| WQHTIDRSGF \| EAKKAEAEVF \| NKNNRWKKRG \| \| 1101 \| VAILPTKFGI \| SFTSKFMNQG \| GALVHVYADG \| SVLVSHGGVE \| MGQGLHTKVI \| \| 1151 \| QVAARAFGIS \| HELVHIEETS \| TNKVPNSQPS \| AASMSTDLYG \| MATLDACEQI \| \| 1201 \| LARLAPVRQR \| LGPDASFSDV \| TNAAYFERVN \| MSAQGFYIIP \| NERCGYDFSK \| \| 1251 \| SVDENIAVGT \| AFNYFTTGVA \| CTVVELDVLT \| GDFHMLSVDI \| LMDLGASINP \| \| 1301 \| AIDIGQIEGA \| FMQGFGLFAL \| EELVWGDNGH \| PWVKRGNLFT \| RGPGAYKIPS \| \| 1351 \| ANDVPLDFNV \| WLESNQKNKF \| AVHSSKAVGE \| PPLFLGSSAF \| FAVKEAIYSA \| \| 1401 \| RADAGHHGYF \| ELRSPVTPER \| ARMACADEML \| KKVFTARGGD \| MVSYQPSGSF \| |
| **S14** | PITG_05074 | \| 1 \| MRHHQRAALI \| LIALACLLAM \| SAAVIDDQRF \| ALNGRTLRTS \| MTSDDEKDDD \| \| --- \| --- \| --- \| --- \| --- \| --- \| \| 51 \| DRMVDLSGLG \| QSAKLTELGA \| LKDIKMKPIA \| ALTLSKLDEA \| GDDLFTSPKF \| \| 101 \| KTWVNYVASV \| AEK**HSTTTMM** \| **SKLTAQYSDG** \| **SLIR**MLEAAK \| KVEGANEIAL \| \| 151 \| RLQQRQVRTW \| IKAEKTADDI \| FELLKLDEGI \| EKLLTNSNLG \| TYVSYMNLFN \| \| 201 \| KYSPGKETTL \| VNTFVTYYGD \| EAVAKTIEA \|  \|  \| |
|  | PITG_23137 | \| 1 \| MRLIAGVLAG \| FLIICEATSA \| SESDHTMAYQ \| TAGTDNTQPT \| DAVENSIASK \| \| --- \| --- \| --- \| --- \| --- \| --- \| \| 51 \| RF**LRADVVMN** \| **RGVERIF**EAL \| KNLLPGAKIF \| AKDGASFSGT \| FVRKMLGDKA \| \| 151 \| FRKQEFERWI \| RSDIDGTALR \| RMLGDMNKKG \| RAELLNRYTA \| FLGSKDGLVY \| \| 201 \| YKPGPIA \|  \|  \|  \|  \| |
|  | PITG_15764 | \| 1 \| MRSCFVLIIF \| LAGICKCLSL \| NWSGNTLKER \| SHTSTNSLPS \| PPFISTIKTP \| \| --- \| --- \| --- \| --- \| --- \| --- \| \| 51 \| KRFLRSYDAP \| KQDNIGHDTD \| ERAGISGIAM \| IDDLAYKWAL \| KNTRDPMDAF \| \| 101 \| QRLHVVKTGG \| KLEGNKEFIR \| WLQYVNRYKA \| TRRVKFGEDE \| LLSLLMKTRA \| \| 151 \| EEELVSLFQS \| LRQYPDITKM \| ASDMQASMIL \| SSASSHRLIN \| EAWLMSRETP \| \| 201 \| GEVFKILRLG \| DNSISRLENN \| PLFIQWLRYV \| TMYRAVHGRI \| WKHYLSVSRL \| \| 251 \| YHNLNFLIQS \| LQNFPDLEK**L** \| **ALSLQTHLYR** \| KWMIEIQLTP \| SELLGLLETT \| \| 301 \| RVARSDPKYR \| NLEAYTMYFA \| ESRGGTPLLN \| KLKTLFTDVD \| PYAALSAASS \| \| 351 \| A \|  \|  \|  \|  \| |
| **S15** | **PITG_13116** | \| 1 \| MARTYLIGGN \| WKCNGTVQSV \| K**DLCALLNK**V \| EITSDKVEVI \| VSPPALHIDL \| \| --- \| --- \| --- \| --- \| --- \| --- \| \| 51 \| AKSLLQKKIA \| VSAQNVSLTG \| TGAYTGEIAA \| EQLVDFGLEW \| TITGHSERRA \| \| 101 \| YYNETDEIVA \| KKTKRALDLG \| LKVIFCIGES \| LEEREGNK**TM** \| **DVLIR**QTQAL \| \| 151 \| ANIVTEADWA \| RIVIAYEPVW \| AIGTGVVATP \| AQVQEAHKDL \| RGWIAGK**VSP** \| \| 201 \| **EVAENVRIIY** \| **GGSVK**GDNCE \| ELIALADVDG \| FLVGGAALKP \| EFEKIIKSAF \| |
| **S16** | **PITG_17261** | \| 1 \| MGLSQERKLE \| YFDK**LESLLE** \| **NYTKIFLVGV** \| **DNVGSAQMQQ** \| **IR**LVLRGR**AE** \| \| --- \| --- \| --- \| --- \| --- \| --- \| \| 51 \| **VLMGK**NTLMR \| KVFNNFVKKN \| PGHPLELFIP \| LLK**GNVGFVF** \| **TNDDLSEIRE** \| \| 101 \| **VLESNR**VPAP \| ARVGSIAPVD \| VIVPPGPTGA \| DPGQTSFFQA \| LQIATKIQKG \| \| 151 \| QIEIVTEVML \| TRKGEK**VGNS** \| **EAALLQK**LDI \| KPFSYGLVIE \| QVYDNGSIFD \| \| 201 \| PAVLDLTEAD \| LCAKFVAGLR \| NVAAMSLELG \| IPTLASIPHS \| IANAFKDLVA \| \| 251 \| IAVECEEFSF \| EKAEPYK**AFL** \| **ADPSAFAVAA** \| **PAGGAAATEE** \| **K**KVEAVEEEE \| \| 301 \| EVDMGGGMDM \| FGGGDEDY \|  \|  \|  \| |
| **S17** | **PITG_09556** | \| 1 \| MAETSIYPRA \| ELDGHNGKAV \| TAIATTR**ENP** \| **NLLLTASR**DK \| SLLVWQLSND \| \| --- \| --- \| --- \| --- \| --- \| --- \| \| 51 \| GEEYGFARRR \| LQGHSHYVED \| VVISSDGQFA \| LSGSWDGTLR \| LWDLNTGITT \| \| 101 \| RRFVGHTKDV \| LSVAFSADNR \| QIVSGSRDKT \| VKLWNTLGEC \| K**YTITEDGHT** \| \| 151 \| **EWVSCVR**FSP \| STANPLIVSC \| GWDKVVKIWN \| LSNCKLR**TNL** \| **FGHEGYLNTV** \| \| 201 \| **TVSPDGSICA** \| **SGGK**DGTANL \| WDLNEGKRLY \| SLVAGDVIHA \| LIFSPNR**YWL** \| \| 251 \| **CAATTSGIK**I \| WDLESK**IVVH** \| **DLQPEIEEPK** \| GKYAQPPHCI \| SLAWSADGSV \| \| 301 \| LFSGYTDGIV \| RVWAVGN \|  \|  \|  \| |
| **S18** | **PITG_02256** | \| 1 \| MLSPRAIQSA \| RGVATKAATG \| KWLASVPMGP \| ADPILGLTDR \| FNKDTDSRK**I** \| \| --- \| --- \| --- \| --- \| --- \| --- \| \| 51 \| **SLGVGAYRDD** \| **DGKPFVLPSV** \| **LEAEK**RIMAA \| GKNK**EYAGIA** \| **GMK**DFVDLSL \| \| 101 \| EFAYGEDCEA \| LKEGR**ITGVQ** \| **TISGTGGVRL** \| **AGEFFNK**FLG \| KNTPVYLPNP \| \| 151 \| TWGNHIPIMK \| NAGMEVR**RYT** \| **YFEPASR**GLD \| FKGLMNDLEG \| APDGSVFLLH \| \| 201 \| ACAHNPTGVD \| PTIEQWQEIA \| DLMKTKKHVP \| FFDCAYQGFA \| SGDASRDAAA \| \| 251 \| IRHFVK**EGHN** \| **IFLSQSYAK**N \| FGLYGERVGA \| LSVVTDSKEE \| AERVQSQLKI \| \| 301 \| IIRPMYSNPP \| IHGSLIVSTI \| LSDAQLKKQW \| YSECKAMADR \| IISMRTALRS \| \| 351 \| AIEKIDATNG \| VQSNWNHITD \| QIGMFCYTGL \| TEAQVAR**MMD** \| **EHHIYLTK**DG \| \| 401 \| R**VSMAGVTTK** \| **NVEYIAKSIT** \| **EVVQNA** \|  \|  \| |
| **S19** | **PITG_14492** | \| 1 \| MVKYVLTGVG \| GNIGGHAADH \| AVEIKKPEDV \| LVLTSSDVSK \| LNPERVAAWK \| \| --- \| --- \| --- \| --- \| --- \| --- \| \| 51 \| AKGAIVEQAD \| YLDVDSLKKV \| FEGAEAIAFI \| STWLIGDGRR \| GQMKNCIKAA \| \| 101 \| KETGVKRVCY \| TSFVGAGSDK \| PNEEVPFLPQ \| DHQFTEQQIY \| ASGLDYNIQR \| \| 151 \| **DYLYQDNTPR** \| FFAQSWHFTE \| DRWLSNSHDV \| PGAYVAISDC \| GRVFAALLLG \| \| 201 \| KQEK**NTVVEV** \| **TGPEAVTDR**D \| MFEWMNSISG \| YK**GQFVDLPD** \| **EELR**AYWLGR \| \| 251 \| GLPTDVYGDF \| SKLPMKLCIG \| DLLCCGEMLA \| NGSMSK**VTDT** \| **VER**LTGRKPL \| \| 301 \| GYKENLLQYK \| EIFPKNQ \|  \|  \|  \| |
| **S20** | **PITG_07671** | \| 1 \| MLSSLSRRSH \| PVVPAIARAS \| SSLMSDVHGT \| THGKKHWKNK \| PLFRRGGDKK \| \| --- \| --- \| --- \| --- \| --- \| --- \| \| 51 \| FR**NGQEAADM** \| **LTDEVIR**RDP \| NQAEYVQAVQ \| NFVNSVVPVF \| DRYPKYAWVM \| \| 101 \| KTLMEPERLI \| QFR**VPWIDDE** \| **GSSR**VNRGFR \| VQFSSALGPY \| MGGLRFHPET \| \| 151 \| THGTAKFLGF \| ETIFR**NALAG** \| **PYGGAHGGSD** \| **FNPMDKSESE** \| **IMR**FCQSYMT \| \| 201 \| ELVNYIGPHT \| DVPTAGVGVG \| PQEIGYMFGQ \| YKRMRQLHPG \| GTEGILSGGA \| \| 251 \| YHYPQVTGYG \| VAHFANRILE \| TRGETLKGKR \| CLISGSGTVA \| LNVARKLLDF \| \| 301 \| GAIPIGMSDN \| FGYVIEDQGF \| SSKSLEELKR \| IKEERNARLG \| AYIMSSTTAN \| \| 351 \| YHPSEEGRLW \| ETPCDYAFPC \| AIQNDVDADA \| VRLLVKNGCK \| GIFEGANFPC \| \| 401 \| TDEAISLIKQ \| HGLAFGPNKA \| SNGGSFALIT \| KNLGSSTQML \| DDEVDVIVKE \| \| 451 \| YMDKLHDRVA \| ASAEEFNAGG \| DLHVGANITA \| FMSVAAAMFR \| QGVV \| |
| **S21** | **PITG_13946** | \| 1 \| MTKSIEEIYG \| EHCIQHIKSV \| DKYDETKLGD \| SRVLSPSIWI \| KSKMHVVGDS \| \| --- \| --- \| --- \| --- \| --- \| --- \| \| 51 \| INYKHSDDGT \| PDFGNDGYTY \| LLQYQFNELD \| KSQMLNNFFF \| KSNEDRESFE \| \| 101 \| QANVECGVEY \| FLATQSYDER \| LYQLCMMIRG \| DLFSNNQNVW \| NLAGFFARQP \| \| 151 \| HSDWHLVRKT \| YLCVLKQKSR \| AFQ**QGLVELT** \| **GRTINDMR**SA \| MVDTSFENDE \| \| 201 \| LEYWAEKYLV \| IENKPNPVDP \| NDNKMRLTCI \| LAVLVAVTFH \| ATGNANSAVA \| \| 251 \| GKASNEYGVD \| MITTTAIFVE \| NPRRKHLFIL \| GADATSQEAF \| HRVVVLHPRG \| \| 301 \| FPLLPRQVER \| QSGWINFEAI \| IDVFLLVHMA \| FCSALTFALL \| AIFTGNTVPS \| \| 351 \| PYTAHSNSTS \| AIFVRKVLD \|  \|  \|  \| |
| **S22** | **PITG_10210** | \| 1 \| MFTSALRASL \| RVQRLGAMKM \| AQSSPAFSSS \| FGLRTFANAT \| AASLGIDK**FG** \| \| --- \| --- \| --- \| --- \| --- \| --- \| \| 51 \| **VTNAK**TQVHR \| **NLSYDEIAVH** \| **EEK**NGEGHFV \| KNGTYTIDTG \| KFTGRSPKDK \| \| 101 \| **FIVDQAPSSK** \| NIWWGDINQP \| VSPEVFDELY \| ETVSEHYGQA \| EK**VYVFDGYA** \| \| 151 \| **GANPASR**KKV \| RFITELAWQH \| HFVTNMFLRP \| QTK**EEIADFK** \| **PDFTIVNACK** \| \| 201 \| VTNKNYKKHG \| LNSEVFVAFN \| IEKDVAVIGG \| TWYGGEMKKG \| IFSMMNYWLP \| \| 251 \| LDGIMAMHCS \| ANKGK**NGDTA** \| **LFFGLSGTGK** \| **TTLSADPHR**Y \| LIGDDEHGWD \| \| 301 \| DEGIFNFEGG \| CYAK**TINLSA** \| **ENEPDIYNAI** \| **K**R**DALLENTY** \| **VDAETKEPDF** \| \| 351 \| **YNTSK**TENGR \| VSYPIYHIPN \| HEPTSSGGHP \| NNIVFLTCDA \| YGVLPPVSK**L** \| \| 401 \| **SDGQAMYHFL** \| **SGYTAK**VAGT \| ERGVTEPTAT \| FSACFGAAFL \| PLHPTKYADL \| \| 451 \| LQKKLQKHNT \| SVYLVNTGWT \| SGGYGVGKRM \| SIKDTR**ACID** \| **AILDGSIKNS** \| \| 501 \| **EFLEDPNFGF** \| **SVPKKLGEIP** \| **ENVLNPR**EAW \| SDKEAYDVTA \| KK**LAGMFQEN** \| \| 551 \| **FK**K**YVSPGVT** \| **DYSK**FGPKL \|  \|  \|  \| |
| **S23** | **PITG_06059** | \| 1 \| MRLTYVLLAA \| ASTLFARHVT \| STPYFANDVA \| LTGVLSLGFI \| HFVGADQSVS \| \| --- \| --- \| --- \| --- \| --- \| --- \| \| 51 \| DQSRFLRGGS \| IDEDDNEEIN \| FLELVKQAMT \| NNFVNKLMKK \| SSFSDLEKID \| \| 101 \| DFGELKRIST \| IIDDKLNNLF \| KQADDA**NKSP** \| **DELAK**ILKEM \| PDVDDALTAK \| \| 151 \| TLEMYTDYLK \| AVGVRVPT \|  \|  \|  \| |
| **S24** | **PITG_10292** | \| 1 \| MLRIVSRFNQ \| PARRVMQTSG \| KSLAGAHNLQ \| CRCGNCVQHP \| TGCTCPRCQS \| \| --- \| --- \| --- \| --- \| --- \| --- \| \| 51 \| RSFSVKSQSH \| YDIPKTQTAV \| VFEDNNAPLQ \| VRKDWPVTQQ \| KDLKPGEVLV \| \| 101 \| RLAYSGVCHT \| DLHVWLGDWP \| LDNKLPLVGG \| HEGSGYVAAI \| GDHSYTNLKI \| \| 151 \| GDPVGVKWLA \| NSCLGCEDCR \| KGHESTCIDA \| DLHGFTVDGS \| FQQWCVSFAD \| \| 201 \| HVTPIPTDLP \| MHAAAPILCA \| GVTVYKALKE \| IGGQCGDFVV \| IPGAGGGLGH \| \| 251 \| LACQYARAMG \| YR**VIAIDSGD** \| **DK**RKLVASYG \| IKDFIDFKEG \| NVKDKVFEAT \| \| 301 \| EGRGAHATVV \| VASGGEAYKD \| ALSFLRPHGA \| VVLVGLPKDT \| YITAEVFGSV \| \| 351 \| LNAHR**IIGSY** \| **VGNR**QDSIEA \| LK**VAAAGDVN** \| **TTYK**IEKLEN \| LPDVFQRMAD \| \| 401 \| GKLAGRIVLD \| **CE** \|  \|  \|  \| |
| **S25** | **PITG_12548** | \| 1 \| MESAPVDDGL \| VFLPQASSGL \| ELLCVALYLS \| LALVAACRAL \| SQGSTFHRSQ \| \| --- \| --- \| --- \| --- \| --- \| --- \| \| 51 \| RSTFQRLMVL \| FAVTRAASFV \| SEGLARNLLN \| RAALCLFFSL \| VLFQTLLWID \| \| 101 \| IANPKVSTRS \| RRIWIAFVVA \| NGLFYAAVLG \| LSVMHEAKVA \| QARHSKSRLN \| \| 151 \| RSTLWTGVLP \| VLFIATGSFV \| SSLGLVYSTW \| KMRHRVERVL \| KPSGNGLRRR \| \| 201 \| LDERVEKKLT \| SALRFTSVVM \| GACSVLFFLR \| TIIYVQRPFS \| HQGCGDIHSP \| \| 251 \| DVCVVVGYVI \| PEIVPCVLFL \| VLMWEVEPTL \| EIPSRRSLSY \| TKGRVTSETT \| \| 301 \| PLLDNEAMPP \| PLLVAAKEFT \| DRIGTASKSA \| VTQPNSPPGS \| QSGYFTVGRR \| \| 351 \| PSPLSLGLGN \| GVLGRARRRL \| ELVVRQHLGG \| ASNEQHSDDP \| WRWPAQSASS \| \| 401 \| TPCAWFSFQC \| FNLTLPSKSS \| VTSSSFVVLH \| LMNAETGAVI \| AEIGRTEISH \| \| 451 \| SEDPCFHMML \| AVDMREQDLL \| RASVYSVRNP \| NSLQNLDSQW \| LVGDPLVPLS \| \| 501 \| SFMTGSLPTI \| GRAAIFSLYS \| PLSRTRSSGE \| IVIRCEAEVK \| GLHAGMSTDG \| \| 551 \| LERITRSFMY \| IGIKSTDAED \| HDDSAYSEIA \| QQMINLASTS \| QRSQGKVLVE \| \| 601 \| EELIESVYTW \| EVPYQLLQLI \| **LSDLLMKLDS** \| **LKR**EIALEDG \| DESSSSTPSS \| \| 651 \| QLSTPSVGGY \| SMPSVITTPR \| ALGTNRVRPA \| TSIGMLSEMI \| IQIQDDAIER \| \| 701 \| KKRKWRLDLV \| KTMEQYISEV \| EDSILRYGAT \| QHAGLTFKPS \| TMKADADLRF \| \| 751 \| LALNLHQQLL \| TVGNAAPTSE \| KDVQYGDQSS \| AARYARLVNS \| FVGTLLSSPL \| \| 801 \| HLSRKASMRD \| QQTDLGSVLF \| VDADKSDENE \| VASPSASEES \| PLEPVEALRR \| \| 851 \| RVISFDSAET \| IVNKRAAGQD \| VSVPSEQRKL \| AETIVAMGIT \| EEEFQRIYGL \| \| 901 \| DENLKKAAMT \| IDTNARNRPP \| FYRHRMYGTT \| TVGAFAAHVY \| GFKSGGVRQM \| \| 951 \| RAELEKLHAR \| LVKEAQASSD \| LTMDALERKY \| NELKWDVERR \| LEVAFCQAMS \| \| 1001 \| ALVTCFQQTL \| YVHTHDHRDF \| GPHPLKACGI \| DYLEMLTRVG \| FLFSAESLLS \| \| 1051 \| TYGNELGMLG \| DTEAAVKELA \| RVHVKLRPVK \| SPRAATFRVS \| ITSGPSGIVI \| \| 1101 \| ELPIITRRAS \| EFPDRSMHRV \| PGQDAVSGAI \| YLPLSTPEQK \| MRAKFLFQKP \| \| 1151 \| IRVIPVIFSQ \| GLNEMQTVAN \| TVGKASLQKE \| INAENVVELE \| SYVKNFAEWV \| \| 1201 \| TKKQCRETSI \| PIYDMEDLDR \| IQTSLVALKL \| SIQLSGRSKR \| MAILSLSSAI \| \| 1251 \| ARSVGGGRVT \| MCKSAKDRTS \| MSITLEEANL \| LVRSHGLLAD \| DMETFTDLLR \| \| 1301 \| TYGVRRENAR \| KNIGKAQYCF \| SALQNYMLPQ \| DYQCPPGTGG \| GSRAYS \| |
| **S26** | **PITG_00585** | \| 1 \| MVSTRSR**TNS** \| **GSSSAAELPQ** \| **K**RSATADASK \| RPTKKSSTPK \| AAASDLAVGK \| \| --- \| --- \| --- \| --- \| --- \| --- \| \| 51 \| PVTVNVTLVN \| QEEKEVVIAD \| TFRDQGVVFF \| MYPRANTPGC \| TKQACGFRDN \| \| 101 \| IQTIKDAGFT \| VYGLSGDSPK \| SLANWKTKQS \| LPFDLLSDPE \| HKLISYFGSS \| \| 151 \| LPGKKVQRSH \| VVVLKGGVVG \| EIAAK**VSPAD** \| **SVSR**AVEFLA \| EGNTVNFDVE \| \| 201 \| LQTDSSATVK \| VQDLFKTRGA \| IFFMYSKADT \| PGCTTQACGY \| NDNLEAINAA \| \| 251 \| GFDVYGLGAD \| SPAELLAWKE \| SQKYKYTFLS \| DPKHHLIGYF \| GSSINDGTRV \| \| 301 \| ERSHVIVLPG \| GKVGQIEHNI \| SPQDSVTKGL \| EFAKSHTVDA \| ASKL \| |
| **S27** | **PITG_00682** | \| 1 \| MLHRALKSSS \| LLMPRVAAAM \| PAQLARASST \| MALPTSLER**L** \| **FENNK**KWREG \| \| --- \| --- \| --- \| --- \| --- \| --- \| \| 51 \| K**KLLDPDYFD** \| **K**TSQGQHPQY \| LWIGCSDSRV \| PAEEITGLAP \| GEMFVHRNVA \| \| 101 \| NLVVSNDISS \| LSVVQYAVEH \| LKVKDIIVCG \| HYGCGGVHAA \| VENKHLGLLD \| \| 151 \| NWLRNIRDVV \| R**IHNDELQEI** \| **DDHEQR**MRR**T** \| **VELNTIEQCI** \| **NVFK**IGLVQR \| \| 201 \| HQVKYGFPR**I** \| **HGLVYDLK**NG \| QLNEMDIDFQ \| SYVRKYQSIY \| K**LHSFPQGEV** \| \| 251 \| **PLR**RSQLQGN \| MIR**ALVEGHE** \| **EEPGHVSAK**F \| VKRAMSK**EPI** \| **LFSDSEINSA** \| \| 301 \| **IAR**AQEGEAN \| NNTVDIEKLA \| RYFDH \|  \|  \| |
| **S28** | **PITG_04081** | \| 1 \| MRSIFYVALA \| FAILCRSSAS \| AALPNLDETR \| **LLSDTSAKR**S \| LRVAGKEVAR \| \| --- \| --- \| --- \| --- \| --- \| --- \| \| 51 \| GGRGEDVVRV \| IVQSSNKIFK \| RPADKDISKL \| IAAAKKAKLE \| KMKEKPSSVV \| \| 101 \| KEVTK \|  \|  \|  \|  \| |
| **S29** | **PITG_03719** | \| 1 \| MAASTHNMKY \| **RFLGNTGLLV** \| **SR**LSFGSWVT \| FDNQLDFEKA \| YSIMEHAYKN \| \| --- \| --- \| --- \| --- \| --- \| --- \| \| 51 \| GVNFFDNAEV \| YANGQSELIM \| GKVVKAGIER \| KVWER**EDLVI** \| **STK**LFFGTKS \| \| 101 \| GPNDVGNSRK \| HIVEGMKASL \| KRFDLDYVDL \| VFCHRPDPAT \| PIEETVRAMN \| \| 151 \| FIIEQGWAFY \| WGTSEWCAK**D** \| **IIEACEIADR** \| LGLIRPVYDQ \| PQYHILERSR \| \| 201 \| VEYDFDVLYK \| KYNYGLTTWS \| PLASGILTGK \| YSKGIPEGSR \| LSMPSYKQML \| \| 251 \| SNGLEEKVAK \| ADKLAEVAKE \| VGCSLAQLSI \| AWVAANPHVS \| TVILGATSIK \| \| 301 \| QLDENLKAME \| FVDKITPELR \| **EKIDAIADFK** \| AK**LVPQAEPH** \| **VINLR**QKWL \| |
| **S30** | **PITG_01904** | \| 1 \| MRFHCTILLA \| ILAFLDSTCR \| AALSSPTMER \| LNVAGNNAAL \| SRRFLRSTGS \| \| --- \| --- \| --- \| --- \| --- \| --- \| \| 51 \| NIGGMAAEER \| SALSKIAGGV \| AK**ISPSGHDI** \| **ANK**MWLKLRT \| NPEVVFKELK \| \| 101 \| LGKTGVK**LDK** \| **ITQLSCGG** \|  \|  \|  \| |
| **S31** | **PITG_05737** | \| 1 \| MYEGMSTPPS \| DKLTQLRPRM \| SDSEAKTMSN \| ESRQSSVSAG \| GHGAPREYDQ \| \| --- \| --- \| --- \| --- \| --- \| --- \| \| 51 \| EDPQRIPMMP \| LMGQGAVASS \| VISRLSVGSH \| LSLNQLATSH \| AGQLRRRPPS \| \| 101 \| TISNKKKSFY \| NAQPNAALRG \| KMPHVPRHWL \| NARFRHNTFF \| ARRLLHTGVP \| \| 151 \| LAFVLAAGLG \| FYYFYKPDTL \| TLNPDMPDVH \| GFTLHSPNLV \| YTFQATSCEN \| \| 201 \| VVGFSALIYH \| TLPAVILLCL \| PGSGWRLFEP \| FKRDEVFSPN \| ASGAPDDDEE \| \| 251 \| DNSNLTKIKR \| RLVFQLCELV \| AVLLLLFQAV \| VVMFFLYMFF \| KGGVFSCSNH \| \| 301 \| SVQLFALVSM \| MCFVWIFTEL \| QYFARFREHV \| KMLLGAFQES \| DQTGDVRTHV \| \| 351 \| MDPGVDQLMN \| QADKALAVVR \| KRLYKATRQG \| DLREMRDILD \| YAEAAGLTSE \| \| 401 \| EQGFPRKSYA \| PASLFLGFFA \| QSRKNPVHVA \| AYHGNIRALE \| LLESRGFDVT \| \| 451 \| AMDKFSRVRF \| STGDLFWYFA \| RFFVARPGVD \| GGGSDEESAA \| SIFRTTLVTP \| \| 501 \| LHCAVSTGQL \| EAVRWLLLRG \| VSARTLARSS \| YRSDRVPPLF \| LAEHPDVVRE \| \| 551 \| LLVHGADPLA \| VPDPGFMNTL \| TALQLAYLRG \| NYAVAQELED \| WGGDVALTPF \| \| 601 \| HSAAGRNDVL \| AVRKFLRKKT \| DVDCL**GELGY** \| **VGLNRRTPLH** \| WAAVSGALEV \| \| 651 \| VDLLVEAGAD \| PNFQDARGRT \| PLHWAARLNR \| TDVLR**SLLDA** \| **GADAKLVDLD** \| \| 701 \| **YMTPMMCA**AS \| GLDATREVFG \| VLTSAGADIN \| YQLPTTGDTA \| LHIAVREENE \| \| 751 \| QSALAILANG \| GNLMKMNIEG \| LRPLDCTQST \| RLLFEIKRAA \| GQRDVMISYT \| \| 801 \| HSHAEFAKKI \| RKALEDANVT \| TWLDLMDPSG \| IGGGSVWREE \| IARGITNAAV \| \| 851 \| VLCLLTEDYA \| QSEWCLKELA \| LAKQVGTPIL \| AVSTEGVGIG \| EDLQVYLYTR \| \| 901 \| QLIPFEPAIT \| QTRRNSTNVR \| QIEYDYDEAK \| FKAQFRLLLD \| GVRDEIEKNR \| \| 951 \| DAVQQKNIAS \| NRRNNGLKQT \| ATGTILATTT \| GGFSRLFQQW \| DPEASGAQKQ \| \| 1001 \| FVFLSHGDKH \| AGFVQQLYRE \| LTDAGVVCYG \| DRNVEGQDFE \| NRIHATQEAI \| \| 1051 \| LRCTCFIVIL \| SKQTMNNELV \| RDQLAFAEDK \| GRPIFPITLN \| DLDPGLDKRY \| \| 1101 \| SLARNELFHF \| MGNGMSFKPS \| ADRLIQGLRR \| HYTAREDNQG \| TMLDNSTRIA \| \| 1151 \| SANTSFVSFT \| FSEPGTKDLD \| REATGEPIIQ \| EGAVLRASSD \| FPRTSSEEPS \| \| 1201 \| NEAESTTVTR \| NFESISGIED \| AQLDLSSTHI \| RM \|  \| |
| **S32** | **PITG_11244** | \| 1 \| MAQSVSGYSV \| GIDLGTTYSC \| VGVWQNDR**VE** \| **IIANDQGNR**T \| TPSYVAFTDT \| \| --- \| --- \| --- \| --- \| --- \| --- \| \| 51 \| ERLIGDAAKN \| QVAMNAHNTV \| FDAKRLIGRK \| FSDPIVQADI \| KHWPFKITAG \| \| 101 \| AGDKPQITVQ \| FKGESKTFQP \| EEISSMVLIK \| MREVAEAFIG \| KEVKNAVITV \| \| 151 \| PAYFNDSQRQ \| ATKDAGAIAG \| LNVLRIINEP \| TAAAIAYGLD \| KKGGERNVLI \| \| 201 \| FDLGGGTFDV \| SLLSIEEGIF \| EVKATAGDTH \| LGGEDFDNRL \| VDHFTQEFKR \| \| 251 \| KHRKDITENQ \| RALRRLRTAC \| ERAKRTLSSS \| AQAYIEIDSL \| FDGIDFNSTI \| \| 301 \| TRARFEDMCG \| DYFRKTMEPV \| EKVLRDAKLS \| KSQVHEVVLV \| GGSTRIPKVQ \| \| 351 \| QLLSDFFNGK \| EPNKSINPDE \| AVAYGATVQA \| AILSGNDSSE \| KLQDLLLLDV \| \| 401 \| TPLSLGLETA \| GGVMTTLIAR \| NTTVPTKKSQ \| TFSTYADNQP \| GVLIQVFEGE \| \| 451 \| RSMTRDNNLL \| GKFNLDGIPP \| MPRGVPQIDV \| TFDIDANGIL \| NVSAVEKSTG \| \| 501 \| KENK**ITITND** \| **KGR**LSQADID \| RMVSEAEKYK \| SEDEANKVRI \| EAKNALENYA \| \| 551 \| YNLRNTLNDE \| KLKDQISEDD \| KKVIDDKVTE \| TINWLDANQS \| AEKEEYEGKQ \| \| 601 \| KELEGVANPI \| LQKMYAAAGG \| APGAEGGMPG \| GMPGGMPGGM \| PGAAGGAPPA \| \| 651 \| GAPDQGPKIE \| EVD \|  \|  \|  \| |
|  | **PITG_03550** | \| 1 \| MAKTPSKKAA \| KTVAKSGKGK \| GKKRVESYST \| YIYKVLRQVH \| PDTGISKRGM \| \| --- \| --- \| --- \| --- \| --- \| --- \| \| 51 \| SIMNSFINDI \| FERIASEAGK \| LSRYNKKSTL \| SSR**EIQTAVR** \| LMLPGELAKH \| \| 101 \| AVSEGTKAVT \| KFTSA \|  \|  \|  \| |
| **S33** | **PITG_16013** | \| 1 \| MPDRLIHNRV \| FKVSKELNRL \| DLLCEQHRRQ \| IGHRKGLPDW \| ERPNTPAGKA \| \| --- \| --- \| --- \| --- \| --- \| --- \| \| 51 \| SGRRGRTPAF \| FAWNEESEHT \| PRPAWQPETP \| QKVARTLHLT \| PTRDRERDED \| \| 101 \| MQPAPERSPY \| ESVGGGNLET \| TTALPSEQVE \| HEGVDETSAI \| EVDSDDRSVE \| \| 151 \| EDRPVVA**ERS** \| **IAVSPLR**EDI \| LDRLHPPAAA \| TVSSRNPTPL \| STVEAASSPY \| \| 201 \| HPPLSPRYRH \| GRRLEASDAA \| VPTPRAQQYA \| AGVTTEQADR \| FRMSRTSGKR \| \| 251 \| FRSPAKRTTL \| WGDLCDTYIH \| QSGASSNQNN \| SRFAHKRATP \| SAAERAFGGS \| \| 301 \| SVFRTLNMNA \| **SAWPTSDSAG** \| **GQRRPVVTGS** \| DAMNVANHAV \| RAFLAGCDDE \| \| 351 \| TKQSEPPMQS \| HLRGGESEWV \| TGAVETSDQL \| PSVSATGSRT \| GNNRKSKRVS \| \| 401 \| **FGGQNTRPQP** \| **RT**LPVLADKT \| TQTEDSLLPA \| RYNVRTLPRD \| TQTGDYPASV \| \| 451 \| RCAACDSAAD \| ESGRPRKIPR \| GSASSIAESP \| AQQHRHRQQL \| ARIYPKYVRL \| \| 501 \| RQGSDIRYRL \| KFTRPPLKGK \| AVTIHVRVIM \| NKIGITASPP \| SLVVTSKDWR \| \| 551 \| QTREITVTSS \| EDSELRTFQI \| HHKIHETYDD \| VYSSAAMLPS \| LFVSVLQKEA \| \| 601 \| TFLFGFGCTV \| DGRLGTDGGT \| NMTTPTPFAC \| RWLHPLQLSC \| GKAHSAIIDV \| \| 651 \| CSNLYCFGLG \| ASGQLGQGED \| SLESSQEPLR \| VPHLASTCVQ \| YVACGSNHTL \| \| 701 \| CLSVDGRVFS \| WGDNSCGQIG \| MGCKTTQIVS \| TPYRVDKIVS \| LRGIVCGGNQ \| \| 751 \| SFILTKTNVL \| ACGSNVAGQL \| GMGDRIDRTS \| FEHIPFFRKV \| WEKLDLEASP \| \| 801 \| LADGTLYAWG \| FGEEGQLGLP \| DEDLEPTRRV \| ALLPVPVHAL \| SGTGATMVSC \| \| 851 \| GGSHTATSLV \| RNSSILQDVD \| AAVLVEQNPV \| LSLYPLL \|  \| |
| **S34** | **PITG_08802** | \| 1 \| MLQVSQFRAL \| RRASATASSS \| RANAFQLTLA \| RAFAAQGGDT \| GVSHEDLQRA \| \| --- \| --- \| --- \| --- \| --- \| --- \| \| 51 \| LDKFQKESAS \| KTVPWFLKNM \| PPSYFRSIEE \| DDRLQHLNAI \| TALVNAQQPE \| \| 101 \| VMLRSTDHRV \| FSHFRSGANF \| PGRLANVLDQ \| LPQTVDGATL \| ARVKIFTSLD \| \| 151 \| DSLGLDIFRF \| GQQEPFLNKT \| EDEQLARASI \| QQFCAGIQAG \| KHVGDSSYPK \| \| 201 \| PGPHFEPEAV \| DTFLKHSNTM \| YVQYSNPRRL \| AWQMELFSQV \| RGTEGVAVDV \| \| 251 \| EHKWETRSDE \| NKLGGGVPQT \| MLTIAASNVI \| PKGFMQKAAT \| YLGLCSLNVV \| \| 301 \| RAHLDVVKDP \| QNGSAHVAMI \| RILVQPSEEA \| QKDHFQFEWS \| KISGNLKYLK \| \| 351 \| WVDDHPVHLT \| LQHPELGLSR \| AELIYAYGNM \| LHGVLAKKDP \| FAYSLTRIME \| \| 401 \| TLEHPQNLPL \| AWRIADFFLT \| KFDPQQERVM \| TDAEQDAVVE \| ELKKEIRRNV \| \| 451 \| EHEDAILLLN \| SMADAVRGTL \| RTNKFVRDRY \| ALSLRMDPKV \| MGYGTVGKDT \| \| 501 \| PFGVFFIYGR \| RFKGFHVRFR \| DIARGGLRMV \| YPSSTDAHAL \| ESARQYNEAY \| \| 551 \| NLAFAQQLKN \| KDIPEGGSKA \| VVLCDPIVGP \| VGDVAPRDFI \| IRKSVKAFSD \| \| 601 \| ALLDLNTTDE \| EVKAKIVDYY \| GKDELIYLGP \| **DENIIPGDIV** \| **WMTKRAAYRG** \| \| 651 \| **YPIP**RAFISS \| KPDAGFNHKV \| YGVTSEGVAV \| FADVALRSQN \| IDPKNQPFTV \| \| 701 \| KITGGTDGDV \| AGNVIKILHR \| EYGDNVHIVG \| ICDGTGVIED \| PQGLDMPELL \| \| 751 \| RLVHESLPLS \| SFDESKVSSK \| GIKHDINTQE \| GIRARNSMHN \| RVKSDLFIPA \| \| 801 \| GGRPNTINEN \| NWRDYLDADG \| KPASGLIVEG \| ANLFITPEAR \| QLLFDNAGVV \| \| 851 \| IVKDSSANKC \| GVVCSSYEIV \| ASMLLETDEF \| LAVKDELVVE \| VVDKLRALAR \| \| 901 \| VEAQLLFREY \| KKDPTSALPP \| ASERISRAIT \| RVHDAVLAHF \| DDVCEEDQQI \| \| 951 \| LFTLIEEHLP \| PKLRELALDR \| VQQNVPLAYI \| RSIVASSLAS \| KIVYREGLQF \| \| 1001 \| TEALPDSNLG \| NMALQYLKQE \| KKVQQLVKDV \| IADLLARGGV \| RSSQLPHKGD \| \| 1051 \| RAGLDTPN \|  \|  \|  \|  \| |
| **S35** | **PITG_01700** | \| 1 \| MYWWPKMYKW \| VGTYVRTRET \| CQWTKSAPST \| AAPLVSLPAP \| KECWQSMSMD \| \| --- \| --- \| --- \| --- \| --- \| --- \| \| 51 \| FVFGLPKDKA \| GNTGIVVLLD \| RLSKMAHLAA \| VSNTIDDDLG \| KEMDAKEQTA \| \| 101 \| IVVRFIAIVL \| YFSVCAGTCW \| AETKQDHVFV \| AGQTLLAASY \| YRVKRRQPLI \| \| 151 \| LGDIQHTLES \| ASCSPSSLLP \| TSTSSVMASS \| DFFREPFTVN \| SKNVVYTDDE \| \| 201 \| ITSQYTYTTT \| RVEGTVATPV \| DEKFTFKTQR \| KIPKLGVMIV \| GLGGNNGSTL \| \| 251 \| VASIIANKHH \| ITWNTK**EGVQ** \| **EPNYFGSVTQ** \| **ASTVR**LGTNA \| NGEGVYIPFH \| \| 301 \| NLLPMVSPNE \| LVIGGWDISS \| LNMAEAMKR**A** \| **MVLDHDLQR**Q \| LVPHLEKIKP \| \| 351 \| LPSIYYPDFI \| AANQADRADN \| LLKGSK**QENL** \| **DAVR**QQIRDF \| KQSNSLDKVI \| \| 401 \| VLWSANTERF \| SDIVEGVNDT \| SANLLESIKA \| GEAELSPSTI \| FAVASILEGC \| \| 451 \| SYINGSPQNT \| FVPGVLDLAE \| EKK**VFVGGDD** \| **FK**SGQTKIKS \| VLVDFLVSAG \| \| 501 \| IKPTSIVSYN \| HLGNNDGK**NL** \| **SAPQQFR**SKE \| ISK**SNVVDDM** \| **VASNR**LLYKE \| \| 551 \| NEHPDHVVVI \| KYVPFVGDSK \| RALDEYTSKI \| FMNGNNTISM \| HNTCEDSLLA \| \| 601 \| SPLILDLVLV \| CELAERITLK \| KEGAAEYEHM \| HSVLSILSYM \| LKAPLVPR**GT** \| \| 651 \| **PVVNALFAQR** \| ECMINIFRAC \| VGLAPESHML \| LENRLASEIN \| ARQ \| |
|  | **PITG_09400** | \| 1 \| MSGALGLRQQ \| GVELESIMRS \| VEGVERKTRI \| FCTLGPSCWT \| EEGLGELIDA \| \| --- \| --- \| --- \| --- \| --- \| --- \| \| 51 \| GMNVARFNFS \| HGDHGSHAET \| LNRLRAALAS \| RPHKNIAIML \| DTKGPEIRTG \| \| 101 \| FLANKDKITI \| KKGSTVELTT \| DYEFLGDETK \| IACSYPELPQ \| SVKVGGSILV \| \| 151 \| ADGSLVLTVT \| EIKEDGVVTR \| **ANNSATLGER** \| KNMNLPGCKV \| MLPTLTEKDE \| \| 201 \| DDLVNFGLVH \| GVDYIAASFV \| RTGQDIDNIR \| QVLGPRGRAI \| KIIAKIESQE \| \| 251 \| GLENFDEILV \| K**TDGIMVAR**G \| DLGMEIPPEK \| VFLAQKMMIR \| KANIAGKPVV \| \| 301 \| TATQMLESMI \| KAPRPTRAEC \| TDVANAVLDG \| TDAVMLSGET \| ANGDYPTEAV \| \| 351 \| QMMSKICVQA \| EGAIHYNELY \| QALHNSVLDT \| YGQMDTQEAI \| TSSAVK**TAID** \| \| 401 \| **INAK**MIVVLT \| ESGNTSASAL \| GGTARQAEGF \| NKGVTARCMG \| SMIGTDSILF \| \| 451 \| RATDLGKQFG \| WVKPGDNVVA \| LHGMVEARSG \| STQHAQGTDC \| GINRQRTHQH \| \| 501 \| AHQGQC \|  \|  \|  \|  \| |
| **S36** | **PITG_07197** | \| 1 \| MTLKNVYIVA \| AKRTPFGNFG \| GKLKDITATD \| LCAHAAKATL \| AAAKLDPALV \| \| --- \| --- \| --- \| --- \| --- \| --- \| \| 51 \| DMVQVGNVAP \| TSPDGAYIAR \| HVQLKAGIPQ \| EKPALTINRL \| CGSGFQSVVG \| \| 101 \| GVQEILLGDA \| EIALCGGAEN \| MSQSPLAVYG \| HQARFGVGLG \| AGLNLQDTLW \| \| 151 \| SALTDSYAK**T** \| **PMGMTAENLA** \| **EK**HNITR**EEC** \| **DAFGLR**SQTR \| WAEAQAAGWF \| \| 201 \| DAELAPLEVK \| AGR**KTEEFAV** \| **DESPR**TVDLA \| KLAKLKPVFK \| KDGVVTAGNA \| \| 251 \| SGISDGAGAV \| LLASEEAVKK \| HNLTPLARIV \| SYQVSGVDPT \| IMGIGPVPAI \| \| 301 \| TGALKRAGLK \| KSDIDIYDIN \| EAFAAQWLSC \| VKELDIDPDI \| VNQSGGAIAL \| \| 351 \| GHPLGASGSR \| ITAHLAHALQ \| RTGKKYAVGS \| ACIGGGQGIA \| LVLENAA \| |
| **S37** | **PITG_23273** | \| 1 \| MVKLFCAIVG \| VAGSAFEVDI \| DQTASVSALK \| KAIKEENAST \| ITCDAKNLQL \| \| --- \| --- \| --- \| --- \| --- \| --- \| \| 51 \| FLAKAGGNAW \| LSSLTEDVKE \| LKKGEKTALV \| KSLTQEEKEL \| QGEDPLSECL \| \| 101 \| EDMDPPKVKQ \| IHVLVVVPGL \| VASTVTIVIE \| EAAGSKPMKE \| LDYYQKCGAS \| \| 151 \| IRKECSDYCC \| EVLDKIDDIY \| DMNELPLPFI \| CVEGSSGMGK \| SQLAFTLQGS \| \| 201 \| RPWFYWHATR \| VTDASQAMYR \| NFKSMSEAFR \| KVVEMDDPVA \| KPMEDILNCQ \| \| 251 \| SGIYQTVDLW \| TCGFISCLLK \| YSKHQSAQMI \| HLEQKIEFRV \| EMRTAQDVYN \| \| 301 \| EVKKMKEENG \| KQLPFFILDE \| MTPNARTSVG \| GKSVAAFQRN \| IFRTCGLVVI \| \| 351 \| VMGTDSKISN \| LVTQATGSST \| GKHMWMSVVP \| KFPPYRFVFD \| PKDQRDQEVW \| \| 401 \| EAAVDWFPVV \| EYIAVNSRGR \| FTRSFVGEIV \| KVLKRAAKQT \| GDGEYSTTDQ \| \| 451 \| VLKGSTTLSD \| LDTSLALSRV \| ELWNLLDEAF \| AGVSRGSQIG \| EVGKAFMDEK \| \| 501 \| DGRYAQLMAI \| SYTNAEETDE \| EPPLKKRRLL \| VGAESMHLHF \| ANLVDEKVTD \| \| 551 \| VIISNGELNM \| EPRTTGPLIP \| WAPQCCFPQM \| DKDVLLYLAI \| LGGKTHSGYY \| \| 601 \| QYTRGGVAHS \| TKFIFSETGA \| FPVHENKNAV \| SNEYKTYENM \| VAHALFCSSR \| \| 651 \| RHGVQGISFD \| DFLECLVGEF \| RDKIWEKVIL \| KDLSGAVCRA \| TELLASYEQR \| \| 701 \| IRDLSTRTMP \| FVAPPNAEWP \| QPIIDMNVRG \| CEFGHLIRAP \| NSERCDIFLD \| \| 751 \| DLGNSNERAM \| FLCECKYRGD \| NVDMGVLKGI \| VAGLNQKWDW \| KIGMLFCPQL \| \| 801 \| ANVRAWTVPD \| VDCMKIDCKT \| **GNVKWVHRPN** \| **AERGAPKVII** \| **VIETGRGGGV** \| \| 851 \| **VG** \|  \|  \|  \|  \| |
| **S38** | **PITG_16048** | \| 1 \| MARTYLIGGN \| WKCNGTVQSV \| K**DLCALLNK**V \| EITSDKVEVI \| VSPPALHIDL \| \| --- \| --- \| --- \| --- \| --- \| --- \| \| 51 \| AKSLLQKKIA \| VSAQNVSLTG \| TGAYTGEIAA \| EQLVDFGLEW \| TITGHSERRA \| \| 101 \| YYNETDEIVA \| KKTKRALDLG \| LKVIFCIGES \| LEEREGNK**TM** \| **DVLIR**QTQAL \| \| 151 \| ANIVTEADWA \| RIVIAYEPVW \| AIGTGVVATP \| AQVQEAHKDL \| RGWIAGK**VSP** \| \| 201 \| **EVAENVRIIY** \| **GGSVK**GDNCE \| ELIALADVDG \| FLVGGAALKP \| EFEKIIKSAF \| |
| **S39** | **PITG_12486** | \| 1 \| MVKSDVYSSP \| IVFSQLDLRV \| GKITEVKAHP \| NSERHYIETV \| DIGKGEELEM \| \| --- \| --- \| --- \| --- \| --- \| --- \| \| 51 \| VMEHQPYFAE \| EELVDRKVVV \| LCNL**KMVKVV** \| **RTRSTGAILL** \| **VANDK**GKVEL \| \| 101 \| LDPSPEAEIG \| ERVYASGEEL \| QDPVTPIQMK \| KNKVWEALCK \| DIKTNNKCEI \| \| 151 \| TYLDRYPVRS \| RAGPVRVESL \| KKVFVTK \|  \|  \| |
| **S40** | **PITG_00160** | \| 1 \| MLLCLCHERS \| RQSIKPRKSD \| RSSRSLSIIR \| TPKVPNASNG \| KTSASSNEVP \| \| --- \| --- \| --- \| --- \| --- \| --- \| \| 51 \| ASPVPPLMRL \| LVKMTSATFE \| RAKGASGIEF \| DVSRDGYGCL \| QIPDINARCA \| \| 101 \| SSTEPATTPS \| LSTFSHSSKV \| WPPPDGYSVM \| AWFR**LDSLER** \| **KDDRERLYRE** \| \| 151 \| **CFMSNTCIHC** \| **RNKIQDECVL** \| **KCSHRACRGC** \| IEALLNSGGE \| CVVCNPPMFY \| \| 201 \| LFRFRSSDGN \| SVSEAFLKGG \| KLYMRTSSNR \| ASAYQFSHTP \| IATKQWHHVV \| \| 251 \| FTHARQRFQP \| SMVSCYLNGV \| LQEHVKISYP \| SGITGSQPLS \| GLLGVPSQAR \| \| 301 \| RCSSAKWMLG \| PFYLLDLPVS \| PPVVNAVFAA \| GPSYDRLFFG \| ATGNNEIGVT \| \| 351 \| FDHLNIPNMV \| MLDSYMWDPV \| RSLIDSVDVE \| RGGRNKLLRR \| SLSLASAASS \| \| 401 \| TAAAIVQDIK \| SSSNVFARIP \| SAAPLVHIPI \| PSERIVVTYS \| ARNGVAKELS \| \| 451 \| MVPSSKLDGR \| PSGHLMGGAT \| LCEAATMADA \| MFDIRSSGCQ \| VAYGLLDEAS \| \| 501 \| TAEEVELALD \| LLRLCMQSNF \| RNLAAMEHDH \| GYGVVNYLLH \| QKASLLSAQC \| \| 551 \| LQTLFRIVGV \| DFELDAPARQ \| SGIEAHPSRD \| SAIRNVQALQ \| YFILDYSLWQ \| \| 601 \| KVPGTDTARL \| LFSTLYSCLA \| RAQLQSMSFI \| RQLLYVLMDP \| ADDEAIRERN \| \| 651 \| TVTDGVSRVV \| VDVILVCLTS \| PSRDSIVESN \| FSDVTSFLAA \| TLSPRFGRYR \| \| 701 \| SEGVDDEVSV \| EVFKSHTLAS \| PTGRLCLSPR \| GSQRMDGNTW \| SEPNDREEKP \| \| 751 \| GPTKVANAQL \| ISHQAKIQEL \| LLDTLVKAVH \| KLDVKESREF \| GEDLDKRGSD \| \| 801 \| ASSGGPLKSV \| TSSSTSSSGA \| ARMQLPSASR \| LTGFRKYLGM \| RWIEYFLFPG \| \| 851 \| DETEFSLRVT \| PSTINAALRL \| LCTLLGNSRY \| ESVFKKEGYY \| RLLAQGLPCN \| \| 901 \| HAMFTTAAVN \| QRFPFQKMWF \| TLFGALLGTP \| VDGVPAAIRL \| EIDYLRKDFE \| \| 951 \| VNIQRDRVVN \| FSILNVIMVL \| LRRHFNDPMA \| MMSSAGDTSM \| VTFVDFDAET \| \| 1001 \| KHSTVLTPST \| EQA**DIYQVEV** \| **LDFLQHIF**EN \| **MPSLHSF**VIS \| GAEKMRQEFM \| \| 1051 \| EELTRLICAA \| ARAYLIEQYP \| HSQAEVSKVL \| EDKQVAVTEY \| NIVVCRAELA \| \| 1101 \| EEAAAVDSNG \| SDPFLHHPVA \| ASGLRLLIAQ \| LMKLLLEAPN \| GSDVIEEYVD \| \| 1151 \| GTANSAVVLP \| PLNSGLVLRF \| QSLVVMGLLD \| HVRAKFDDDD \| IVAKHKHFGA \| \| 1201 \| NVREFVKFVV \| AKMHSWQHPQ \| HGDGCPAVFA \| CCGAAHFVGG \| PSRLLEMVLF \| \| 1251 \| VLADAHIGAC \| GVNGSGLSSL \| STPSSFGGML \| SEKLSKGKKR \| KPFRQLMDRM \| \| 1301 \| TRSVELDTLV \| SELYIALNAV \| ILHVFHGRGA \| EVGDEELEAM \| LQQIHLHRDV \| \| 1351 \| VFGSQSSHDK \| RFLGCLCRYL \| LQLLSDANVR \| PLQEAAAHLW \| IDLMLFQRSF \| \| 1401 \| IDDLLTVEIR \| KSGAPPYSVN \| LMKNGFDVLL \| ECADAPAEKR \| IVQSSSFATF \| \| 1451 \| SKWLELVGPP \| LKELEGNLDR \| IYIHFVVETK \| EAVHETWTAY \| HKKANHRKSK \| \| 1501 \| YEKQFDARYD \| WFVSMENAYI \| ESLLRSQQNE \| FRRQLKWEQD \| RVDRQKFIAN \| \| 1551 \| RSRYDLESSS \| PRRYSWRLDF \| TEGPYRMRKR \| LTQMAQTLEP \| SCRPGLLQRR \| \| 1601 \| CSESDVNLRT \| VSSQAKSCKT \| AEPLAKRQQF \| DESETGNTRQ \| PATKRAFATS \| \| 1651 \| ASSSAVLSSR \| ERLRKGSFEA \| LFTKYVKNER \| RPSQSFRFNR \| LSAGHDSIVE \| \| 1701 \| HDDHEEEKAG \| DVSGVESSAV \| FESAVNSGGV \| AMEDVVDEKL \| RPLLMPGDEI \| \| 1751 \| IAIYDCLRID \| GMDSSPGVFL \| LCNDHVYIVD \| NYQRQSQPFV \| SSHNDIGSEH \| \| 1801 \| NSQTRVTEVP \| QGSTTLLERR \| LSWRLHESPH \| HSQSVARSRD \| THQCRFWAYE \| \| 1851 \| DITELHKRRY \| QLRHVALEFF \| ANDGRNYLVT \| LESLEQRELV \| LHALLAKCPN \| \| 1901 \| VQGAASGLDG \| VSGGGDLYSQ \| LRKLLRNSMT \| ERWVQGDISN \| FAYLMHLNTL \| \| 1951 \| AGRSYNDLTQ \| YPVFPWVLAD \| YDSEILDLSD \| PIVYRDLRKP \| MGALQREEEF \| \| 2001 \| RARYDGLLES \| LGVADDAASD \| HALSSRPFHY \| GTHYSSAAIT \| LHYLMRLEPF \| \| 2051 \| TSHFRRLHGG \| KFDHADRLFT \| SIVGAWKSAA \| GFEGAQNGTQ \| DVKELIPEFY \| \| 2101 \| YLPEFLENVN \| ACVFGTSQTG \| VVVGDVELPP \| WANGSPTEFV \| RLNRAALESP \| \| 2151 \| YVSANLHHWI \| DLIFGYKQQG \| PAAVEACNIF \| YHLTYEGSVD \| LDAITDASTK \| \| 2201 \| RAILDQITEF \| GQTPSQLFRT \| PHPVRAVAAS \| TSGNIATNSS \| LFGGSLGPSS \| \| 2251 \| GPSYAQGESG \| DGRRVATSPL \| TARAALASSF \| LEGGEIISRM \| QTMLSSGPVL \| \| 2301 \| SGTFVGTTEA \| TFSPVLETSA \| LLQQEPRRQV \| PVNPLLAFYR \| RGQQGSTSGS \| \| 2351 \| NTNIHNIAWT \| SGGVAREEKV \| VVAGPKCLLI \| PPRNNEYLAW \| GFHDQSVKVV \| \| 2401 \| STSSAEIGGH \| GSESKVIACL \| ELDVEIDVAT \| ITTDGRIVIT \| SSPSLPVLRV \| \| 2451 \| **WRFNSTRRSL** \| **AASLAAASGS** \| **SSASATSSSA** \| **AAVATSLAAA** \| **SALSAPHRRR** \| \| 2501 \| **TYTTMP**SSTR \| SLTLMGSVST \| PMHRNRITAL \| QASRAYSVLV \| SGCAGGVAVL \| \| 2551 \| WDLNRRRFIR \| QLPPICGQDD \| TRVRGITTIC \| INEVTGDIVV \| AAGSTFGVYN \| \| 2601 \| INGVLRVRLD \| DSVVVRANGV \| LRST \|  \|  \| |
| **S41** | **PITG_20172** | \| 1 \| MVKLFCSIVG \| VAGSAFSVEV \| DEGKTVDDLK \| EAIKAKKAND \| FKEVDADKLQ \| \| --- \| --- \| --- \| --- \| --- \| --- \| \| 51 \| LFLAKKDEGR \| GPWLTEEEVK \| KDVIDTTGLK \| LLGAARARLR \| RVGLSDVDVG \| \| 101 \| GVDEDEEVEG \| RGPVNVLVVV \| PEQDGTISKE \| MFAATTPLTL \| EQVEMSMNKV \| \| 151 \| LRERDEKASA \| YSFSDLNTAM \| EEQIVKKMRL \| TENIPDVKEP \| VDTSIAGYSW \| \| 201 \| IPKIVESEES \| QRAGYMAYLQ \| QHLKTLMDRG \| DFLLDDIAGD \| KSVLNIVDPR \| \| 251 \| LPFAMKGTAD \| VLLINRTSKN \| PLIKLAGVSL \| VIELKKKVEP \| GHVPQAIGQL \| \| 301 \| VLCSMKAPLN \| CYPLSLLTDL \| NDHWHFSWFS \| DKHVLTQLTL \| KYPKNAFRFI \| \| 351 \| EAAVLGRTES \| APPPPSFMPG \| SFKIIKVDDF \| LPQPVDARAE \| **EMMKRYELMA** \| \| 401 \| **DVVEPEFLMA** \| **RRMDYARQLV** \| **QSMP**MYSYMY \| T \|  \| |
| **S42** | **PITG_00827** | \| 1 \| MAARFLNRVA \| GIGATIGFGG \| FCLQECIYDV \| DGGHRAVIFD \| RKDGILDKSV \| \| --- \| --- \| --- \| --- \| --- \| --- \| \| 51 \| GEGTHFKIPF \| FQYPTILDVR \| SNYRLISSRT \| GTK**DLQNVNI** \| **SLR**CLYRPNA \| \| 101 \| DKLSHIYAEY \| GPDFADR**ILP** \| **SVGNEVLK**SI \| VAQYDAVELL \| ARRDQVSIQI \| \| 151 \| AKEMNDRCRN \| FFLLLDDVSI \| THLEYGPEFT \| RAVEQK**QVAQ** \| **QDAER**QKFVV \| \| 201 \| MRSEQERKAA \| VIKAEGESEA \| AR**LVSDAVSK** \| SGSGFIEVQR \| IDAAR**EIAET** \| \| 251 \| **LAK**SRNVTYL \| PNQEGGNGGV \| LLGLQ \|  \|  \| |
| **S43** | **PITG_14221** | \| 1 \| MGQDSAYVVV \| LYYKYVRLCE \| TREELKAFAA \| AHDQLCSSLG \| VTGRVRLALE \| \| --- \| --- \| --- \| --- \| --- \| --- \| \| 51 \| GINGTLGGSS \| ANVQSYIDTM \| KQQPQFADVD \| WKTSSSRVEP \| FPELHVRVVA \| \| 101 \| EIVALELPDD \| AYDLSLRGKH \| LTPEQFRSEQ \| LSSDAESIAL \| IDVRNTYEFN \| \| 151 \| VGHFEGALNP \| KTRRFGQFPQ \| WVRDELPMLQ \| QKDKVLMYCT \| GGIRCEKASA \| \| 201 \| YLKHLGLENV \| YQLEGGIHRY \| LERFPDGGGL \| FQGKNFVFDQ \| RVTVASEDKT \| \| 251 \| VTGQCERCQV \| PHDTLPGTRC \| AYCRMHVLLC \| ESCRESAKAR \| GETDEDVFCD \| \| 301 \| EHSPLVSGSL \| GDLETRFEVL \| QDSLSNEQGR \| GKKGRRRSLR \| K**QLDTVERR**I \| \| 351 \| QRLATA \|  \|  \|  \|  \| |
| **S44** | **PITG_10334** | \| 1 \| MRRSALRRPE \| SLTDKYRILG \| KIGEGTYGMV \| FKAESHSAAL \| LPRPTSGGSN \| \| --- \| --- \| --- \| --- \| --- \| --- \| \| 51 \| AVEDEEPMTF \| AIKLVKSHKE \| GKNDVVLSSA \| TVREIKLLRE \| MHHDNVMHLH \| \| 101 \| DVHVDPRDKS \| LALVFEYGDH \| DLHDIIVQSK \| QKPL**GEYTRK** \| **SLMYQILK**GV \| \| 151 \| DYMHDVWVMH \| RDMKPQNILV \| VGHGRKRGQV \| KLGDFGLARI \| FKEPIKALSD \| \| 201 \| VERVVVTLWY \| RAPELLLGAK \| HYTKAVDLWA \| VGCIFVELIN \| TRELFCGKEV \| \| 251 \| EGSNAPFQKD \| QLDKIFKVLG \| MPTPQTWEGL \| ENLPEYNHVV \| QMGRERKYPT \| \| 301 \| QSELKNAVKV \| GPGRAGAALL \| DLLSRLLEYD \| PITAKEALEH \| EYFKEIVRSL \| \| 351 \| VCLFRGLV \|  \|  \|  \|  \| |
| **S45** | **PITG_01470** | \| 1 \| MATRDVTRQF \| LQLRAEEKAK \| VLRRKNIVSH \| REEGNALIKS \| ADQESTSVAI \| \| --- \| --- \| --- \| --- \| --- \| --- \| \| 51 \| APGWVDVVNG \| TNQHVARIK**E** \| **MMEKLNK**LHT \| SRLMVRFDGQ \| ESKYEREIDQ \| \| 101 \| LTQDITDEFR \| SAEKGLRRMA \| QSDRDGEFSA \| ADAKTRQNVQ \| RALATQLQTL \| \| 151 \| SGDFRKSQKT \| YLARVKNQKE \| GPVEFDFLAE \| NDAKQKRRGG \| ADTGFTQAQI \| \| 201 \| TEVEIAEDVI \| NE**RDQEIQRI** \| **ATSI**TELATI \| FKELAVLVID \| QGTILDRIDY \| \| 251 \| NMEQVVEQTE \| KGIEELEKAE \| ETQKNSRPMK \| CIGLLLVLIF \| AMTLLLVLKH \| \| 301 \| S \|  \|  \|  \|  \| |
| **S46** | **PITG_17907** | \| 1 \| MVANNANSSA \| NNGTCPTAAW \| HPPTDAESTT \| FRHQTSLPKL \| PIPDLEGTCQ \| \| --- \| --- \| --- \| --- \| --- \| --- \| \| 51 \| RFLASVKALQ \| TPEEQHQTER \| SVK**EFLASDG** \| **PR**LHQQLLDY \| DKDKHSYIED \| \| 101 \| FWYEAYLNHR \| SSVVLNVNPF \| FVLEDDPTPS \| RNNQLSRAAS \| LVLGALKFVN \| \| 151 \| ALRRGTLEPD \| MWRGNMALCM \| HQYKRLFGCA \| RVPSDGADDV \| MVDELSKHIV \| \| 201 \| VVCRNQFYWF \| DVIWEDGVTA \| ITERELLANL \| KAINEDALKT \| DDVEAASNAV \| \| 251 \| GVLTTERRAK \| WANLRTKLQA \| KNKDTLAVID \| RALFLVCLDT \| TSPPDAAAFA \| \| 301 \| ATALHGTYSI \| TKDGVQTGTC \| MNRWYDKLQL \| IVCENGVAGC \| NFEHAFVDGH \| \| 351 \| **TVLRFVSDVF** \| **TDTIIRFAQT** \| **IRAGNYA**FLE \| ASYRAPQLSP \| SDTPGRPRVQ \| \| 401 \| PHKLEWELDH \| ELQEGIKFAE \| AQLTDLMLQN \| EVKVLEFTAF \| GKLFITQHNM \| \| 451 \| SPDAFVQMAF \| AAAYYFQYGS \| APCIYEPVLT \| KRFLHGRTEA \| ARAMTADALE \| \| 501 \| FVETFFSEKT \| PLEKIGSLRK \| AISTHVANVR \| DCAGGKGPER \| HLYALECLWQ \| \| 551 \| HEKAADNSKP \| TPALFADDAW \| CKLNHSVLST \| SNCGNPSLRL \| FGFGPVVPDG \| \| 601 \| FGIGYIIKDE \| GIQFCASSRH \| RQTERYLKNL \| ESYLLRVQDL \| LMYEEEIRFP \| \| 651 \| HSTAAKKKAV \| QSTFKGYGFF \| DDGGQPTEVK \| ALASIGAVGK \| PLV \| |
| **S47** | **PITG_03685** | \| 1 \| MLPSRLSSFV \| RRCASTSARN \| SFVDRLRNEL \| AEIEAAGTYK \| NER**VIASPQG** \| \| --- \| --- \| --- \| --- \| --- \| --- \| \| 51 \| **AK**ISVNGDTV \| LNFCANNYLG \| LSNHPAVEKA \| AADTLKERGF \| GLSSVRFICG \| \| 101 \| TQDIHKQLED \| AISAFHGTED \| TILYPSCFDA \| NAGLFEAVLN \| KEDAIITDEL \| \| 151 \| NHASIIDGIR \| LCK**AERHRFK** \| **HMDMNDLEQK** \| LKDTQHCRTR \| LIATDGVFSM \| \| 201 \| DGDVAPLQDI \| MALAEKYDAQ \| LFIDECHATG \| FFGPTGRGSD \| EYCGINGKVD \| \| 251 \| VINSTLGKAL \| GGSTGGYTTG \| RKEVVDLLRQ \| RSRPYLFSNS \| LGPSVVGASL \| \| 301 \| KVFEMLTESS \| EFVDKIRANT \| HHFRDR**MTAA** \| **GYTLK**GSRDH \| PIAPVMLGDA \| \| 351 \| RLASELADDM \| LKRGIYVIGF \| SYPVVPKGQA \| RIRVQLSAAH \| SIEDVDKAVD \| \| 401 \| AFIECGKARG \| VIN \|  \|  \|  \| |
| **S48** | **PITG_06595** | \| 1 \| MLSRVGLTAM \| RGARSMATDA \| KKVTGSVGQI \| TQIIGAVVDV \| QFKDNLPPIL \| \| --- \| --- \| --- \| --- \| --- \| --- \| \| 51 \| NALEIKTQDN \| TRIVLEVAQH \| LGENTVR**TIA** \| **MEGTDGLVRG** \| **QECVDTGNPI** \| \| 101 \| **MVPVGPETLG** \| **RIMNVIGESV** \| **DER**GPINAKR \| HAPIHTSAPL \| LTEQGSGAEI \| \| 151 \| LVTGMK**VVDL** \| **LAPYAK**GGK**I** \| **GLFGGAGVGK** \| TVVIMELINN \| VANNHGDQRR \| \| 201 \| RHDHGSK**AAL** \| **VYGQMNEPPG** \| **AR**ARVGLTGL \| TVAEYFRDVE \| GQDVLLFVDN \| \| 251 \| IFRFTQACSE \| VSALLGRIPS \| AVGYQPTLAT \| DLGALQERIT \| STKKGSITSV \| \| 301 \| QAIYVPADDL \| TDPAPATTFS \| HLDATTVLSR \| **QISELGIYPA** \| **VDPLDSK**SRM \| \| 351 \| LDPR**IIGQEH** \| **YDVAR**ATQK**L** \| **LQDYK**GLQDI \| IAILGMDELS \| EDDKLTVARA \| \| 401 \| RKVQKFMSQP \| LHVAEVFTGK \| AGK**FVPLTDT** \| **ISSFK**DIIAG \| NYDDLPEAAF \| \| 451 \| YMVGGIEEVK \| AKAADLAAEL \| DE \|  \|  \| |
